# A high-resolution functional network-organized atlas of human superficial white matter from ultra-high-field diffusion MRI

**DOI:** 10.1016/j.isci.2026.116671

**Published:** 2026-07-06

**Authors:** Yifei He, Yu Xie, Hiuying Yip, Yoonmi Hong, Ye Wu

**Affiliations:** 1 School of Computer Science and Technology, Nanjing University of Science and Technology, Nanjing, Jiangsu 210094, China; 2 Tsinghua Shenzhen International Graduate School, Tsinghua University, Shenzhen, Guangdong 518055, China; 3 Department of Psychiatry, University of North Carolina at Chapel Hill, Chapel Hill, NC 27599, USA

**Keywords:** superficial white matter, diffusion MRI, tractography, white matter atlas, functional networks

## Abstract

Superficial white matter (SWM) supports local cortico-cortical communication. Still, its whole-brain organization remains difficult to characterize *in vivo*, due to its short length, high curvature, proximity to the gray-white matter interface, and individual variability. Here, we constructed a high-resolution, tractography-derived human SWM atlas using 7T diffusion MRI data from 171 participants in the Human Connectome Project. We combined deterministic and probabilistic tractography, multi-stage clustering, geometric filtering, and a deep-learning classifier trained on expert-informed SWM labels to identify anatomically plausible SWM clusters. The resulting atlas retained approximately 10% of whole-brain streamlines and comprised 643 and 1,403 SWM clusters under Yeo 7- and 17-network parcellations, respectively. Cross-dataset analyses supported reproducible SWM-like tractography patterns. We further provide network-level annotations, Neurosynth-based functional associations, and a TW-dFC-derived uncertainty index as complementary references for interpreting clusters. Together, this work provides a publicly available SWM atlas and processing framework for future studies of white matter connectivity.

## Highlights

• Geometric and network classifiers identify plausible SWM clusters
• Yeo-organized SWM atlas contains 643 and 1,403 clusters for Yeo-7 and Yeo-17
• TW-dFC uncertainty captures cluster-level structure-function organization
• Neurosynth meta-analysis links SWM clusters to cognitive associations

## Graphical abstract

**Figure:**
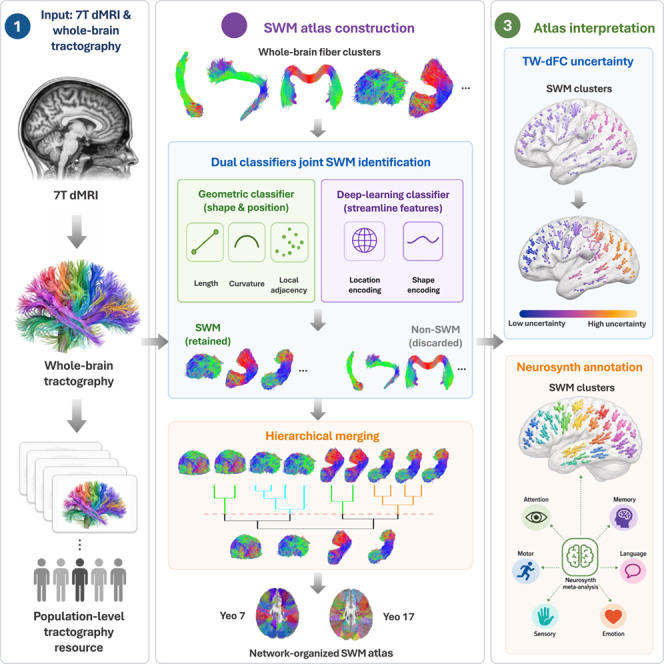

## Teaser

Biological sciences; neuroscience; neuroanatomy; techniques in neuroscience

## Introduction

The superficial white matter (SWM) of the human brain comprises myelinated axonal fibers located near the cortical surface, forming short-range corticocortical connections between adjacent gyri.^1^^,^^2^ It comprises most of the white matter volume, facilitates communication in higher cognitive functions such as learning and decision-making, and has a higher neuronal density than other white matter systems.^3^ SWM myelinates later than other regions and has been linked to various cognitive processes and neuropsychiatric conditions, underscoring its importance in clinical research and diagnosis.^4^^,^^5^^,^^6^^,^^7^^,^^8^

Diffusion magnetic resonance imaging (dMRI) provides a non-invasive method for mapping brain white matter fibers, thereby facilitating the study of SWM structure and its relationship to various diseases.^5^^,^^9^^,^^10^^,^^11^ However, tracking SWM pathways is challenging due to their shorter lengths, complex trajectories, and substantial inter-individual variability, which complicate reconstruction efforts. While fiber atlases are commonly used to extract specific tracts from whole-brain tractography, current SWM atlases are limited by their reliance on a single tractography method, small sample sizes, and a lack of biological validation, which affects their generalizability. Additionally, existing datasets typically focus on anatomical information and do not incorporate functional data, hindering a comprehensive understanding of SWM connectivity in the brain. These challenges underscore the need for a more comprehensive mapping of SWM to understand its structural and functional roles better.

To address the challenges of understanding the structural and functional organization of SWM, we present a high-quality SWM atlas derived from a finely parcellated whole-brain atlas, along with a comprehensive analysis of its functional connectivity. (a) We introduced a fine-grained SWM atlas featuring over 600 well-parceled SWM clusters, developed from a whole-brain tractography atlas with 33,256 anatomically defined fiber clusters. (b) We applied the same processing pipeline to construct separate SWM datasets for the 7- and 17-network parcellations based on the Yeo atlas, enabling researchers to choose the atlas that fits their functional granularity needs. (c) We designed a multi-criteria SWM filter combining geometric feature thresholding with a deep learning classifier to identify thousands of consistent SWM clusters. Hierarchical clustering merged SWM clusters with similar geometric characteristics, resulting in 643 merged clusters for the 7-network and 1,403 for the 17-network parcellations. (d) Finally, we developed a methodology integrating structural and functional information, called tract-weighted dynamic functional connectivity (TW-dFC), to investigate the functional anatomy and topographical organization of SWM. This approach enabled us to characterize structural-functional uncertainty across SWM clusters and provide complementary references for interpreting their relative reliability.

## Results

### A finely parcellated SWM atlas with high individual-level reproducibility

**Figure 1:**
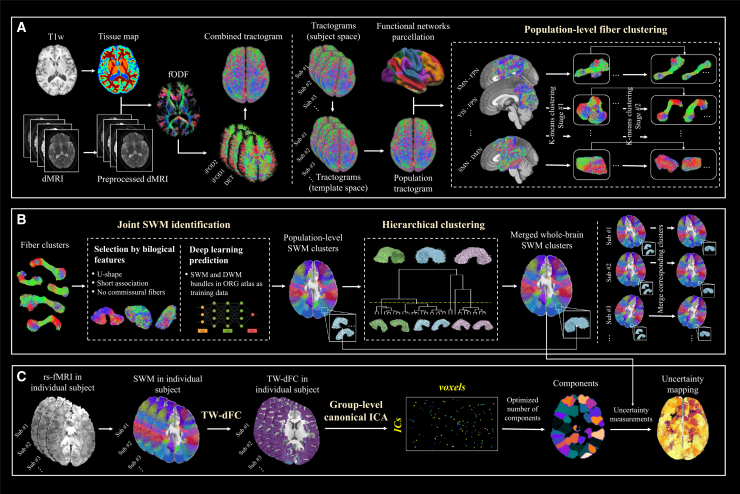
Overall pipeline (A) Population-level tractography reconstruction and two-step fiber clustering. (B) Population-level SWM cluster identification. (C) Uncertainty measurement of SWM clusters based on TW-dFC and group-level ICA.

We first constructed a population-level fiber cluster dataset based on the HCP 7T ultra-high-field cohort (Figure 1A). To identify SWM from the finely clustered whole-brain tractogram and construct an atlas, we then estimated the joint probability of each fiber cluster belonging to SWM using two complementary approaches: shape- and position-based criteria, and deep learning classification (Figure 1B). This approach yielded thousands of filtered SWM clusters, with an average cross-subject consistency of over 99.6% across 171 subjects in both the 7- and 17-network parcellations. Here, cross-subject consistency was interpreted as a measure of individual-level reproducibility within the study cohort, indicating that most group-defined SWM clusters could also be identified in the vast majority of individual subjects.

To further assess the reliability and cross-dataset reproducibility of the proposed pipeline, we applied it to two independent high-quality dMRI datasets, including one ultra-high-resolution sample^12^ and one ultra-high-gradient sample.^13^ After extracting SWM pathways from these datasets, we generated track density images (TDIs) using the tckmap function in MRtrix3 and performed min-max normalization. We then quantified the pairwise similarity among the atlas-derived SWM TDI and the TDIs from the two external datasets using SSIM,^14^ LPIPS,^15^ and PSNR.^16^ As summarized in Table 1, the SWM TDIs showed consistently high similarity across datasets, suggesting that the proposed pipeline can produce broadly reproducible SWM-like spatial patterns across independent high-quality dMRI acquisitions.

**Table 1:** TDI similarity evaluation

Metric	Dataset	Ours	High-res	High-grad
SSIM	Ours	–	0.91	0.92
	High-res	0.91	–	0.92
	High-grad	0.92	0.92	–
LPIPS	Ours	–	0.23	0.28
	High-res	0.23	–	0.28
	High-grad	0.28	0.28	–
PSNR	Ours	–	31.13	33.37
	High-res	31.13	–	30.83
	High-grad	33.37	30.83	–

Image similarity metrics between the TDI image of our SWM atlas and the TDI images of SWM extracted by our SWM identification pipeline from the high-resolution and high-gradient samples. From left to right, the similarity is quantified using SSIM, LPIPS, and PSNR, respectively.

**Figure 2:**
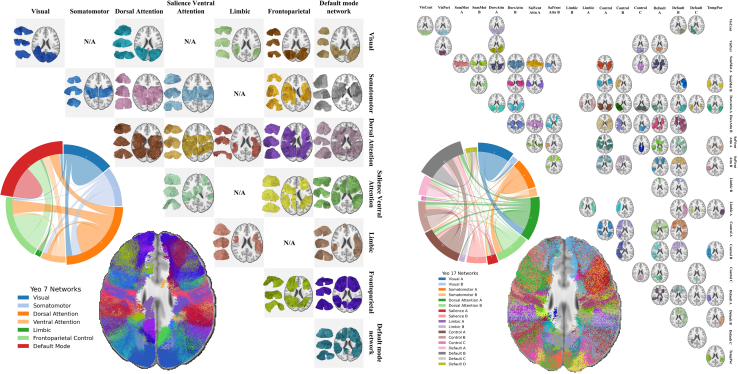
SWM clusters visualization The SWM atlas was constructed from fiber clustering and categorized based on the functional brain networks connected by the fibers. Two versions are provided: one based on the 7-network parcellation (left) and the other on the 17-network parcellation (right). Blank regions indicate the absence of SWM pathway connections between the corresponding functional areas. Circular connectome diagrams illustrating the strength of fiber connections between functional brain regions, based on parcellations into 7 and 17 functional networks. The width of each outer ring segment is proportional to the volume of the corresponding functional region.

Subsequently, we applied hierarchical clustering to merge similar SWM clusters, thereby minimizing redundancy from a large number of highly similar clusters (Figure 1B). In identifying and merging the SWM pathway, we deliberately avoided using manually predefined thresholds. Instead, we adopted adaptive strategies to determine optimal parameters, thereby reducing dependence on manually selected parameters. This included selecting the number of clusters in whole-brain fiber clustering, utilizing the *Z* score of joint probability as the criterion for SWM pathway identification, and establishing the optimal merging distance threshold in hierarchical clustering based on the elbow point of the Davies-Bouldin (DB) index. Ultimately, this process yielded 643 SWM clusters under the 7-network parcellation and 1,403 clusters under the 17-network parcellation, representing the most fine-grained tractography-derived SWM atlas to date. Figure 2 shows the resulting SWM fiber clusters for both the 7- and 17-network parcellations, with circular graphs illustrating the streamline-count-based connection strength between functional networks. Although we attempted to use adaptive strategies wherever possible, some basic parameters were still manually specified based on previous studies, such as the window length for TW-dFC estimation. The sensitivity of these parameters was not systematically evaluated in the present study. Therefore, the potential influence of these parameter choices should be considered when interpreting the results or applying the proposed pipeline to other datasets.

### SWM clusters show reproducible network-level patterns and descriptive functional associations

From whole-brain tractography, 1,719 SWM clusters (62 million streamlines) were identified for the 7-network parcellation and 4,189 clusters (63.5 million streamlines) for the 17-network parcellation, classified by connected functional networks. Most clusters showed high cross-subject consistency (>90%), with only a few exhibiting low consistency, suggesting potential false positives.

**Figure 3:**
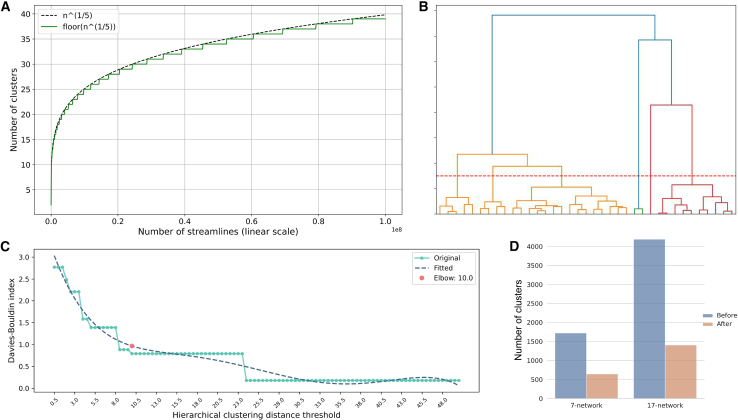
Hierarchical merging diagram (A) Variations in the number of clusters with the number of fibers (number of fiber streamlines from 100 to 10^8^), where the black dashed curve illustrates the computed fifth root values, and the green segmented line denotes the resulting cluster numbers after flooring. (B) Hierarchical clustering schematic. Fiber clusters with distances below the threshold are merged. (C) Elbow-based automatic clustering threshold selection. (D) Number of SWM clusters before and after hierarchical clustering.

After hierarchical clustering, SWM clusters were reduced to 643 and 1,403 for the 7- and 17-network parcellations, respectively (Figure 3D). Analysis of functional connectivity revealed that for the 7-network parcellation, 65.2% of clusters formed inter-network connections, while 34.8% were intra-network. Clusters most frequently connected to the dorsal attention network (41.7%), followed by the default mode (31.9%) and frontoparietal control (31.3%) networks, with only 4.2% linked to the limbic network. Additionally, 52.7% of clusters were right-lateralized. For the 17-network parcellation, 76.3% of SWM clusters formed inter-network connections, while 23.7% were intra-network. The most frequently connected regions were FPN-A, DAN-B, and DAN-A (19.5%, 19.5%, and 19.4%, respectively), whereas LIM-B was the least connected (0.1%). Slight right lateralization was observed, with 54.4% of clusters located in the right hemisphere.

**Figure 4:**
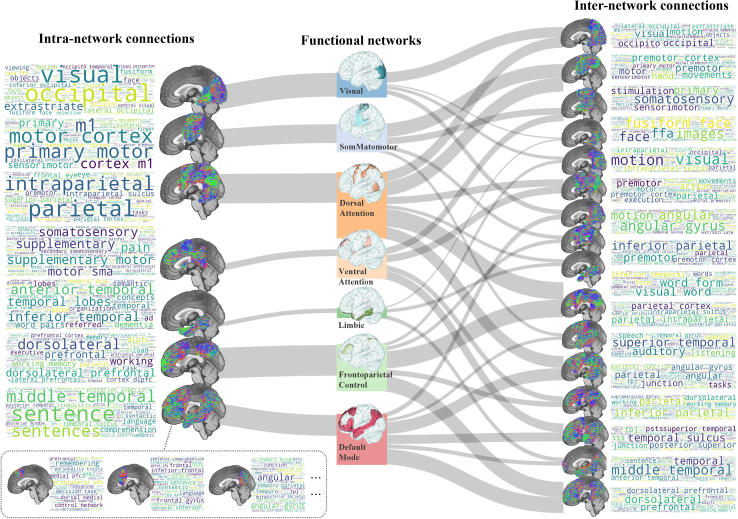
Neurosynth Sankey diagram The Sankey plot illustrates intra- and inter-network connections across the seven functional networks, with the corresponding word clouds highlighting their most strongly related cognitive functions.

To further elucidate the cognitive functions associated with each SWM cluster, we employed the Neurosynth platform https://neurosynth.org/, a tool for large-scale automated meta-analysis of published neuroimaging studies. Briefly, Neurosynth extracts activation coordinates from the neuroimaging literature, identifies high-frequency terms in individual articles, and performs voxel-wise meta-analysis. Figure 4 depicts the intra- and inter-network fiber connections, with the corresponding word clouds highlighting the cognitive functions most closely associated with these fibers. The word clouds of connections shown in the figure are obtained by integrating analyses across all fibers associated with each region pair. In addition to the network-pair-level results shown in the figure, we also computed meta-analysis results for each SWM cluster. These cluster-level results are available in our online resource. Representative word clouds for three SWM clusters connected to the default mode network are shown in Figure S4, illustrating that functional terms were computed at the individual-cluster level and that clusters connected to the same functional network pair may still be associated with different functional terms.

These results suggested that our SWM clusters could support more fine-grained functional investigation beyond network-level categorization. Even SWM clusters that connect the same pair of functional networks can exhibit different functional associations, suggesting that cluster-level analysis may reveal additional functional specificity. At the same time, the substantial correspondence between the meta-analysis results and the functional networks connected by the fibers was consistent with our extraction pipeline, providing supportive evidence for the plausibility of the identified SWM clusters.

### Density-based analysis reveals variability and network-specific patterns in merged SWM clusters

To better elucidate the architecture of the SWM cluster after hierarchical clustering, a density-based statistical analysis was performed. The density of the merged clusters was defined as the average number of original clusters per merged cluster, and the corresponding variance was also calculated. A higher density indicated that more original SWM clusters were grouped into the same merged cluster during hierarchical clustering, suggesting greater similarity and redundancy among the original clusters. In contrast, the variance reflected how much this merging pattern varied across clusters within the same network-pair category. This density metric also helped indicate whether the SWM clusters associated with a given functional network pair were relatively homogeneous or heterogeneous, thereby providing additional insight into how SWM was distributed across different functional network connections.

In the 7-network parcellation, the three merged fiber categories with the highest densities connected the dorsal attention and limbic networks (density = 4.43, variance = 5.29), the Visual and Limbic networks (density = 4.33, variance = 5.33), and the intra-network connection within the Limbic network (density = 4.00, variance = 4.50). Conversely, the three merged cluster categories with the lowest densities connected the somatomotor and default mode networks (density = 1.93, variance = 0.78), the ventral attention and frontoparietal control networks (density = 2.00, variance = 0.87), and the visual and frontoparietal control networks (density = 2.07, variance = 0.84).

We also computed the merged cluster densities and variances under the 17-network parcellation. The three cluster categories with the highest densities connected dorsal attention A (DAN-A) and default mode C (DMN-C) (density = 5.00, variance = 9.00), default mode A (DMN-A) and default mode C (DMN-C) (density = 4.69, variance = 3.56), and dorsal attention A (DAN-A) and limbic A (density = 4.27, variance = 8.62). In contrast, the three cluster categories with the lowest densities connected limbic B and default mode B (Default-B) (density = 1.00, variance = 0.00), visual peri (VisPeri) and default mode A (Default-A) (density = 1.20, variance = 0.20), and frontoparietal control C (FPN-C) and default mode C (DMN-C) (density = 1.33, variance = 0.33). Notably, the original fiber cluster connecting limbic B and default mode A (DMN-A) consisted of only a single cluster and was therefore excluded from the density analysis. In general, the variance of fiber connections was proportional to their density, with higher-density connections corresponding to larger variances. However, the fibers connecting dorsal attention B (DAN-B) and frontoparietal control C (FPN-C) exhibited a notably larger variance despite a relatively low density (density = 3.50, variance = 7.32).

### Uncertainty characterization of SWM clusters based on structural-functional decomposition

**Figure 5:**
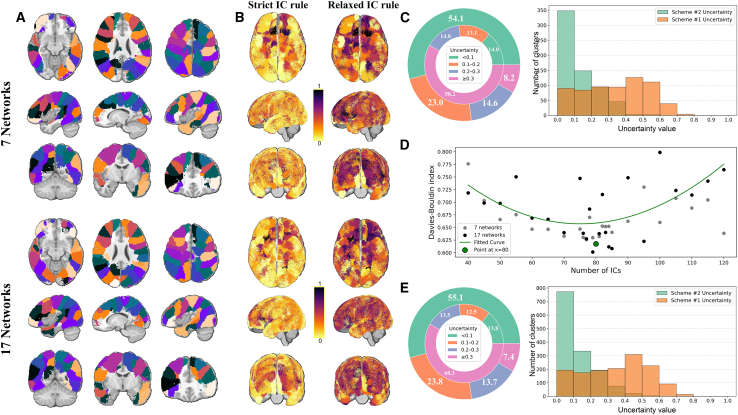
TW-dFC-based uncertainty (A) 80 ICs of resting-state TW-dFC of SWM, obtained through IC analysis (ICA) for the 7- and 17-network parcellations. (B) Uncertainty mapping of SWM clusters under two different uncertainty measurement schemes, for the 7- and 17-network parcellations, respectively. (C) Proportions of SWM clusters categorized by different uncertainty levels under the 7-network parcellation. The circular graph shows only the proportion of clusters with uncertainty values less than 0.3. The inner and outer rings represent the strict and relaxed IC rules for uncertainty measurement, respectively. (D) Illustration of the procedure used to select the optimal number of ICs based on the DB index. (E) Proportions of SWM clusters categorized by different uncertainty levels under the 17-network parcellation. The circular graph shows only the proportion of clusters with uncertainty values less than 0.3. The inner and outer rings represent the strict and relaxed IC rules for uncertainty measurement, respectively.

To assess the structural-functional uncertainty of SWM clusters, we performed a voxel-level decomposition of SWM using ICA of TW-dFC images, yielding 80 independent components (ICs). Based on this parcellation, we quantified the uncertainty of each SWM cluster by examining the extent to which its fibers traverse multiple ICs. Figure 1C illustrates the procedure for ICA-based decomposition and uncertainty mapping. Under the relaxed IC rule (allowing fibers to span up to two consecutive clusters), the majority of clusters exhibited low uncertainty. Specifically, in the 7-network parcellation, 91.6% of clusters (590/643) had uncertainty values below 0.3, with 77.1% below 0.2 and 54.1% below 0.1. Similarly, in the 17-network parcellation, 92.6% of clusters (1,299/1,403) showed uncertainty values below 0.3, with 78.9% and 55.1% below 0.2 and 0.1, respectively. In contrast, under the strict IC rule (requiring fibers to remain within a single component), the proportion of low-uncertainty clusters decreased substantially. For the 7-network parcellation, only 41.8% (269/643) of clusters had uncertainty values below 0.3, with 27.1% below 0.2 and 14.0% below 0.1. Similarly, for the 17-network parcellation, the proportions decreased to 39.7% (557/1,403), 26.2%, and 13.8%, respectively. The distributions of uncertainty values under both rules are illustrated in Figures 5C and 5E, where the outer ring represents the relaxed IC rule and the inner ring represents the strict IC rule for the 7- and 17-network parcellations, respectively. Overall, the uncertainty distributions were highly consistent across the two network configurations, indicating that most SWM clusters exhibited broadly similar descriptive annotation with respect to the underlying structural-functional decomposition.

### Low-uncertainty SWM clusters more often originated from intra-network connections

To further investigate the distribution of uncertainty in SWM clusters, we examined the functional cortical networks most frequently connected by fiber clusters with the highest and lowest uncertainty under the relaxed IC rule for both parcellation configurations. The results of the 7-network parcellation indicated that low-uncertainty fibers (bottom 10% in uncertainty) originated more from intra-network connections within the dorsal attention network (21.88%), the default mode network (10.94%), and the somatomotor network (10.94%). In contrast, high-uncertainty fibers (top 10% in uncertainty) primarily originated from connections between the frontoparietal control and default mode networks (20.31%), the ventral attention and default mode networks (9.38%), the somatomotor and dorsal attention networks (9.38%), and the somatomotor and default mode networks (9.38%). For the 17-network parcellation, low-uncertainty fibers also commonly came from intra-network connection within one functional network: the somatomotor A (SMN-A) network (9.29%), the dorsal attention B (DAN-B) network (9.29%), and the default mode A (DMN-A) network (7.14%), whereas high-uncertainty fibers were more frequently from connections between the frontoparietal control B (FPN-B) and default mode C (DMN-C) networks (6.43%), the frontoparietal control A (FPN-A) and default mode C (DMN-C) networks (5.00%), and the somatomotor A (SMN-A) and dorsal attention B (DAN-B) networks (5.00%).

## Discussion

This study provides a new tractography-based resource for investigating SWM in the human brain. We identified a comprehensive collection of anatomically plausible SWM pathways by constructing a highly detailed SWM tractography atlas to date and utilizing ultra-high-field dMRI data. This approach, which integrated multiple tractography algorithms (see Table 2 for specific tracking algorithms and parameter settings) and employed a multi-criteria filtering process, enabled the identification and clustering of the SWM pathways based on their morphological and spatial characteristics. To further investigate the structural and functional distribution of SWM, we employed TW-dFC. This integrated imaging framework combines structural and functional information, followed by ICA, to quantify the structural-functional consistency index of the SWM pathways and to highlight pathways with high uncertainty. This work provides a tractography-based resource and an analytical framework for characterizing the anatomical and structural-functional organization of SWM, which may benefit future studies on brain connectivity and disorders.

**Table 2:** Tractography parameter settings

	SD_STREAM						iFOD1						iFOD2					
ACT	*✓*	*✓*	*✓*	*✓*	*✓*	*✓*	*✓*	*✓*	*✓*	*✓*	*✓*	*✓*	*✓*	*✓*	*✓*	*✓*	*✓*	*✓*
RK4	*✓*	*✓*	*✓*	*✓*	*✓*	*✓*	*✓*	*✓*	*✓*	*✓*	*✓*	*✓*	–	–	–	–	–	–
Dynamic seeding	*✓*	–	*✓*	–	–	–	*✓*	–	*✓*	–	–	–	*✓*	–	*✓*	–	–	–
Grid seeding	–	*✓*	–	*✓*	–	–	–	*✓*	–	*✓*	–	–	–	*✓*	–	*✓*	–	–
WM/GM interface seeding	–	–	–	–	*✓*	*✓*	–	–	–	–	*✓*	*✓*	–	–	–	–	*✓*	*✓*
Angle	60	60	75	75	60	75	15	15	30	30	15	30	45	45	60	60	45	60
Step size (× voxel size)	0.25	0.25	0.25	0.25	0.25	0.25	0.25	0.25	0.25	0.25	0.25	0.25	0.5	0.5	0.5	0.5	0.5	0.5

Parameter sets for tractography reconstruction.

### Current SWM atlases and their limitations

In recent years, with the rapid advancement of dMRI-based fiber tractography techniques, the SWM pathway has garnered increasing attention. As a commonly used resource to extract the SWM pathway from whole-brain tractograms, various SWM atlases have been developed to analyze the group-level SWM structures across individuals,^2^ which are listed in Table 3. Nevertheless, existing SWM atlases exhibit several limitations that may compromise their completeness: (i) all the existing SWM atlases were constructed through a single fiber tracking algorithm, which may limit the range of reconstructed pathways and increase the risk that some plausible pathways are not captured. Studies have shown that the SWM pathway identified via deterministic tractography exhibits lower reproducibility across subjects than that identified via probabilistic tractography,^23^ highlighting the sensitivity of SWM pathway identification to the chosen tractography methodology; (ii) current SWM atlases were typically generated from datasets with limited size (usually fewer than 100 subjects), and most reconstructions were performed using 3.0T dMRI data, which may constrain the accuracy and generalizability of SWM pathway tract delineation compared to data collected with higher-resolution or advanced acquisition schemes with increased angular and spatial resolution, as well as higher signal-to-noise ratio;^25^^,^^26^^,^^27^ and (iii) the number of SWM pathway bundles identified in current atlases is generally limited. At the same time, the delineation of most SWM bundles lacked clear biological validation, thereby reducing the interpretability of analyses across different SWM pathways. The scarcity of high-quality, large-sample SWM datasets has significantly limited research on SWM compared with DWM. At the same time, investigations on the functional connectivity of SWM are also scarce. Existing studies on the function of SWM have primarily focused on quantifying the associations between microstructural metrics of SWM pathways and certain disorders.^28^^,^^29^^,^^30^ In contrast, analyses of the overall distribution of SWM functional characteristics remain lacking. To address these challenges, the primary aim of this study is to develop a high-quality SWM pathway dataset that enables researchers to obtain a finer-grained segmentation of the SWM pathway with additional functional interpretation.

**Table 3:** Summary of SWM atlases

Study	Tracts	Atlas population	Field strength
Oishi et al.^17^	21 blade-like regions with 4 short association tracts	81 healthy subjects	1.5T
Guevara et al.^18^	36 DWM tracts and 94 SWM bundles (47 in each hemisphere)	12 NMR subjects	1.5T
Guevara et al.^19^	100 U-fiber bundles (50 per hemisphere)	79 healthy subjects	3.0T
Román et al.^20^	93 SWM bundles (44 in the left hemisphere and 49 in the right hemisphere)	74 healthy subjects	3.0T
Zhang et al.^21^	58 DWM tracts and 198 SWM clusters (16 categories)	100 HCP healthy subjects	3.0T
Guevara et al.^22^	Not given	897 HCP healthy subjects	3.0T
Román et al.^23^	525 SWM clusters	100 HCP healthy subjects	3.0T
Li et al.^24^	77 SWM bundles	755 HCP subjects	3.0T
Proposed atlas	643 (or 1,403) SWM clusters	171 HCP subjects	7.0T

### The proposed fine-grained SWM atlas composed of clusters

**Figure 6:**
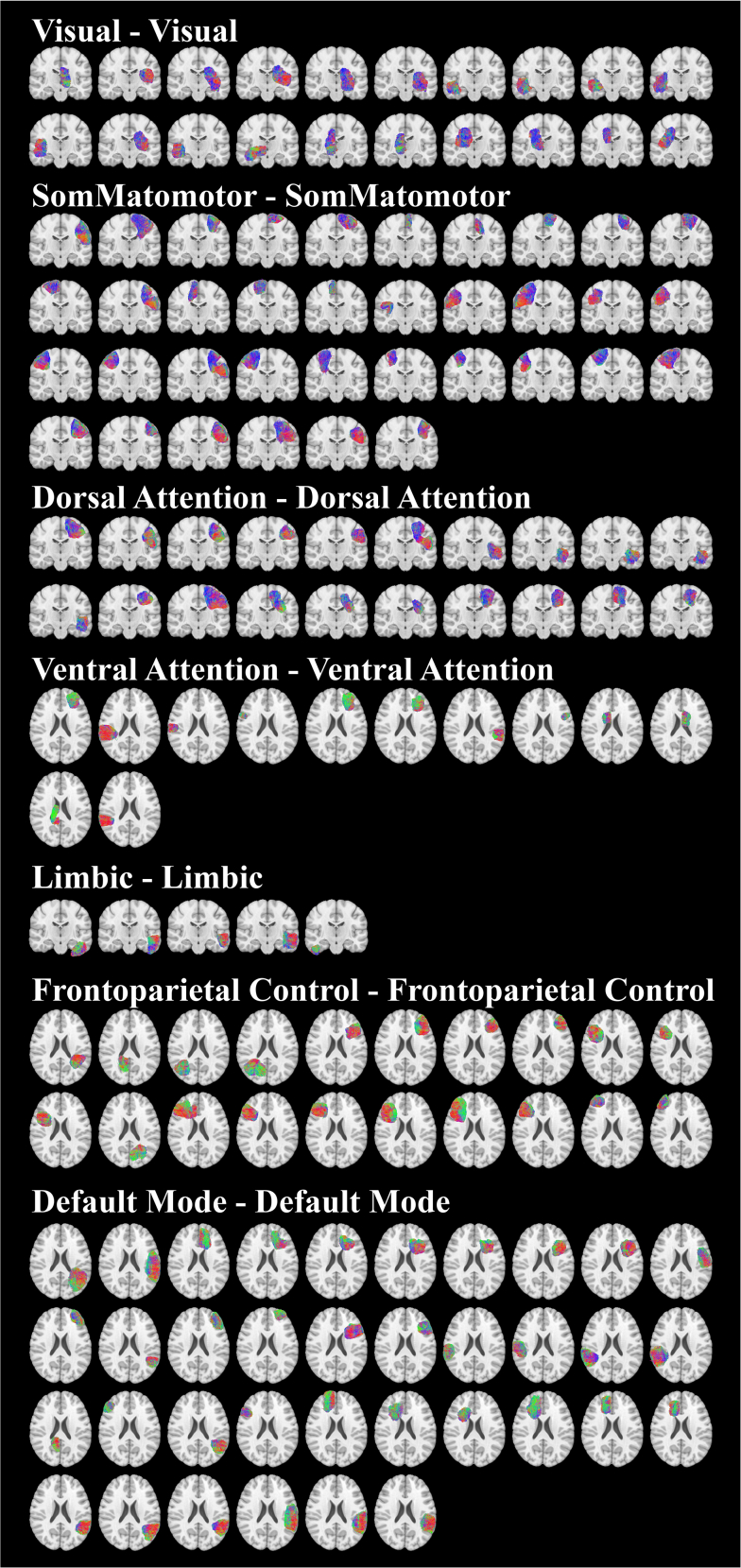
Intra-networks SWM clusters The illustration of intra-network SWM clusters after hierarchical clustering.

**Figure 7:**
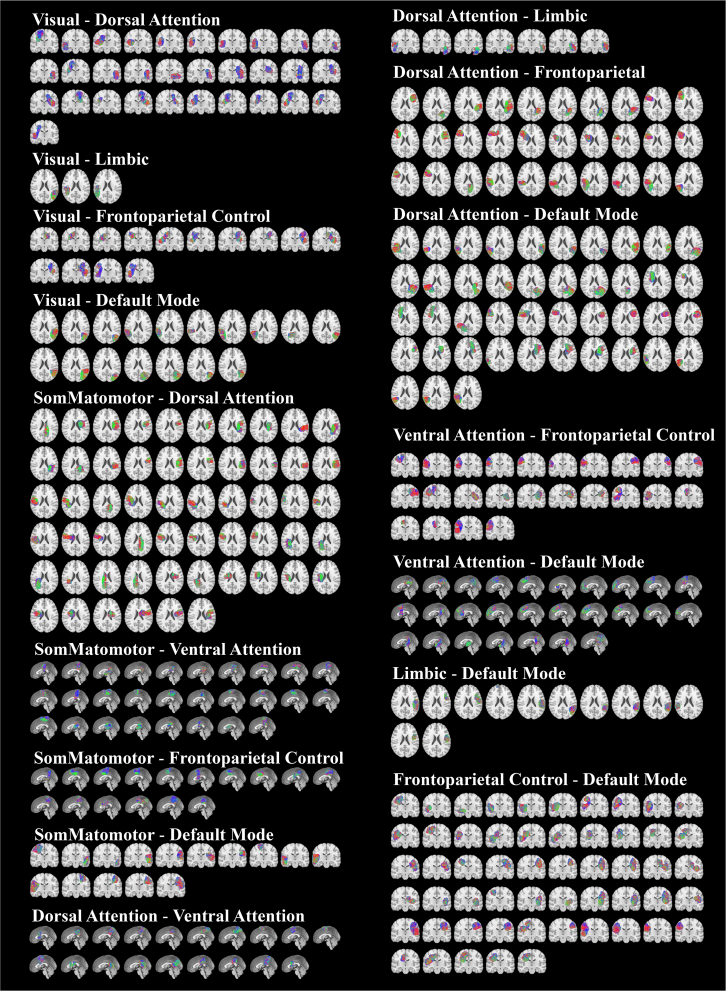
Inter-networks SWM clusters The illustration of inter-networks SWM clusters after hierarchical clustering.

The SWM atlas generated in this study represents a more comprehensive resource for SWM characterization than previous resources, constructed through a multi-step filtering pipeline that integrates anatomical and deep learning-based probability measures to improve consistency and anatomical plausibility. The resulting SWM clusters display diverse morphological characteristics, suggesting that SWM pathways are not limited to short, U-shaped configurations. In total, 643 and 1,403 SWM clusters were identified under the 7- and 17-network parcellations, respectively, providing a detailed resource for future studies of SWM organization. The 643 SWM clusters under the 7-network parcellation are illustrated in Figure 6 (intra-network connections) and Figure 7 (inter-network connections).

The number of SWM clusters was relatively small compared to the total number of whole-brain fiber clusters, representing 5.2% (1,719 of 33,256) under the 7-network parcellation and 6.4% (4,189 of 65,184) under the 17-network parcellation. The proportion of SWM clusters among all whole-brain fiber clusters is noticeably smaller than the proportion of SWM fibers among all whole-brain fibers. Specifically, under the 7-network and 17-network parcellations, the SWM pathway accounted for 10.1% (62.1M of 613.0M) and 10.0% (63.5M of 636.6M) of all fibers, respectively. This discrepancy may suggest that the SWM pathway exhibits greater spatial and morphological concentration than deep white matter fibers, with many fibers grouped into the same clusters, reflecting greater similarity in their spatial trajectories and morphological patterns. The slight differences in fiber counts between the 7- and 17-network parcellations likely arise from fibers located near network boundaries. These boundary-crossing fibers may exhibit sensitive connectivity patterns and thus represent valuable targets for studying fine-grained SWM organization.

Our proposed fine-grained atlas of SWM fibers can contribute to the advancement of current tractography research.^2^ SWM data organized at the level of fiber clusters can facilitate targeted analyses of local SWM morphology and enable more precise computation of microstructural metrics at the scale of small SWM clusters. This scale differs from conventional tract-based definitions. Moreover, our SWM atlas can serve as a useful large-scale training dataset for deep-learning approaches explicitly designed for SWM.^31^ It may also help redirect analyses that are currently performed at the whole-brain level toward the SWM specifically, enabling more focused modeling of short-range cortico-cortical pathways and their structural and functional variability.^32^^,^^33^

At the same time, as the proposed SWM atlas is derived from tractography, it also carries the inherent limitations of tractography-based reconstruction. It should therefore be interpreted with appropriate caution. In particular, given the known limitations of current tractography methods, including their relatively low specificity, some identified SWM clusters may still contain false-positive streamlines. To support interpretation and downstream use, we provide additional measures, including cross-subject consistency and the TW-dFC-based uncertainty metric, which can help users assess the relative reliability of individual SWM clusters according to their specific research purposes. We want to clarify that the primary goal of constructing this SWM atlas was to provide a sensitive and comprehensive resource for population-level SWM mapping. Further biological and anatomical validation in future work may help refine the identified SWM pathways and improve the atlas’s overall specificity.

In addition, the initial clustering granularity in our framework was adaptively determined from the large-scale population tractogram of the current cohort to achieve a sufficiently fine-grained SWM subdivision. Although this strategy was suitable for the present dataset, its applicability to smaller cohorts or datasets with fewer fibers remains to be systematically evaluated in future independent reliability analyses. Moreover, since the current atlas was constructed from a specific acquisition setting, its direct transferability to cohorts acquired at different field strengths (e.g., 1.5T or 3T) or with different spatial/angular resolutions may be affected by differences in image contrast and tractography quality. For practical use, we recommend treating the 7T-derived atlas primarily as a high-resolution reference space. When applying the atlas to 3T data, users should avoid assuming one-to-one recovery of all fine-grained clusters, especially small or highly curved SWM clusters near the gray-white matter interface. Instead, the atlas may be more appropriate for group-level comparison, coarse network-level annotation, or as a prior for SWM localization after dataset-specific tractography and quality control. Future applications of the atlas or the proposed pipeline should account for potential variations across datasets with heterogeneous acquisition protocols.

### Hierarchical clustering of SWM pathway: Higher cluster density accompanied by greater variance

By calculating the density of clusters after hierarchical clustering (i.e., the number of original SWM clusters merged into a single cluster) and the corresponding variance, we observed that higher cluster density was generally associated with greater variance. A higher density indicates greater similarity or compactness among fiber clusters. Notably, fibers connected to the limbic network tended to exhibit higher cluster density. In addition, we found that connections with high cluster density (e.g., DAN-limbic, visual-limbic, and limbic intra-network connections) contained fewer fiber clusters than other network pairings. This may suggest that the SWM pathway, with smaller overall numbers—such as those connecting to the limbic network—exhibits lower within-cluster dispersion but greater between-cluster variability.

The functional role of the SWM pathway connecting the limbic network remains to be further investigated. Previous studies on the clinical relevance of SWM have suggested that these fibers may be associated with specific disease mechanisms. For instance, Bigham et al.^34^ reported significant alterations in microstructural metrics of the SWM pathway connecting the Limbic network in patients with Alzheimer’s disease. Liu et al.^35^ found that temporo-limbic SWM connections are related to the pathological mechanisms of temporal lobe epilepsy. Although existing evidence is still insufficient to fully explain the distinctive characteristics of the SWM pathway connecting the limbic network observed in our study, these findings collectively highlight the potential research value of limbic-related SWM and point to a promising direction for future investigations.

### Meta-analysis results were closely related to the connected functional networks

The meta-analysis results from the Neurosynth platform revealed close associations between the identified fiber clusters and functional brain networks. The three terms most closely associated with intra-network connection fibers in the visual network were the occipital, visual, and extrastriate areas, all of which are associated with visual processing. Moreover, cognitive functions associated with fibers that link different networks were consistent with potential interactions among these networks. For example, the connection between the visual and dorsal attention networks was particularly relevant for motion and dynamic visual processing. Interestingly, fibers connecting the dorsal attention and limbic networks showed strong associations with word form and visual word processing, suggesting their possible involvement in reading and language comprehension. These findings may inform future investigations into the roles of SWM. Additionally, examining fiber clusters could reveal previously unexplored cognitive associations, thereby providing additional insight into possible functional associations of SWM. Future research might also compare cognitive associations of SWM and DWM connections within the same networks to highlight functional distinctions.

### SWM pathway categories based on functional network parcellation

In this study, we categorized the SWM pathway by the cortical regions it connects to and used this classification as the basis for subsequent clustering and analysis. Since the functional brain network atlas we adopted includes two parcellation schemes—dividing the cortex into 7 and 17 functional networks, respectively—we constructed two versions of the SWM atlas accordingly. We conducted functional analyses for both, resulting in two sets of experimental outcomes. This design serves three primary purposes. First, we aimed to develop an SWM atlas that integrates both structural and functional information to facilitate future functional studies of SWM. To this end, we adopted the widely used Yeo atlas^36^ as the basis for classification and atlas construction. Second, we intended to ensure that the resulting SWM pathway clustering is robust and stable and not overly sensitive to the initial parcellation scheme. The consistency between the two atlases supports the observed network-level patterns. Lastly, while the overall distribution patterns between the two schemes are similar, finer-scale discrepancies may reflect inherent differences introduced by the parcellation scheme itself. The SWM pathway, which shows divergent classifications between the two schemes, is likely located near network boundaries and may represent more sensitive or functionally heterogeneous regions. They may thus be of particular interest for further functional investigations.

### Structural-functional uncertainty of SWM clusters

Previous studies have demonstrated the close relationship between brain structural and functional organization, often integrating these features to study connectivity,^37^ cognition,^38^ and disease.^39^ In this study, we applied TW-dFC, a voxel-level representation that integrates structural information from fiber tracts and functional information from fMRI. Based on the TW-dFC representation, we further applied ICA to obtain a voxel-wise decomposition of SWM regions, which was then used to quantify the uncertainty of each SWM cluster.

Under this framework, fibers within the same SWM cluster were expected to traverse as few IC categories as possible. Based on this assumption, we quantified the uncertainty of each SWM cluster and compared the proportions of clusters falling within different uncertainty ranges. Figures 5C and 5E show that the uncertainty distributions derived from the 7-network and 17-network parcellations were highly similar, indicating that this measure is relatively robust to the choice of network configuration. In addition, allowing clusters to traverse two ICs (relaxed IC rule) substantially reduced the overall uncertainty compared to the strict IC rule, suggesting that most SWM clusters maintain a relatively coherent annotation under this criterion.

We analyzed which brain regions were connected by SWM clusters with the highest (top 10%) and lowest (bottom 10%) uncertainty, finding that low-uncertainty fibers originate primarily from intra-network rather than inter-network connections. In contrast, high-uncertainty SWM pathways predominantly link different functional brain networks. Under our definition of uncertainty, SWM clusters connecting regions within the same functional network tend to exhibit greater coherence, which may relate to their known microstructural properties (e.g., thin axons and low myelination, which support efficient short-range communication^40^). This finding is also broadly consistent with prior observations that SWM networks strongly correspond to the cortical functional networks they underlie.^41^

It is worth noting that this computational approach has certain limitations, and basic structural properties of the SWM clusters may partially influence the resulting uncertainty measure. For example, smaller clusters are more likely to be concentrated within the voxels of a single IC, thereby exhibiting lower uncertainty. However, small clusters may also exhibit characteristics that increase uncertainty, such as being located near the boundary between two ICs or containing too few streamlines to provide stable, uniform sampling of the underlying signal. Conversely, larger clusters can sometimes still be largely encompassed within a single large IC and thus remain relatively consistent. Therefore, the relationship between uncertainty and cluster size is not strictly linear.

To further assess this issue, we performed an additional control analysis using univariable and multivariable linear regression models with several simple structural factors, including streamline count, spatial span, and mean streamline length. Additional methodological details and visualization of the regression results are provided in the supplementary material (Table S1 and Figure S3). All of these variables showed significant associations (*p* ≤ 0.001) with the uncertainty metrics, although their explanatory power remained limited. Specifically, streamline count was negatively associated with uncertainty, whereas spatial span and mean streamline length were positively associated with uncertainty. Under the strict IC rule for uncertainty measurement, the univariable models explained only 3.3%, 10.0%, and 14.4% of the variance in streamline count, span, and mean length, respectively, and 18.3% jointly in the multivariable model. Regarding the relaxed IC rule, the corresponding univariable explained variances were 2.6%, 14.6%, and 18.5%, and the multivariable model explained 22.9% of the variance. These findings suggest that the uncertainty metric is not merely a surrogate for simple size-related or geometric properties of the clusters, although they partially influence it. We therefore emphasize that this measure should be regarded as a descriptive index reflecting the relative functional consistency or uncertainty of fibers within the identified SWM clusters. In contrast, its precise biological meaning and its potential associations with other properties of the fiber clusters still require further validation in future work.

### Identification of SWM pathway remains challenging

SWM plays a crucial role in brain connectivity and has been implicated in various neurological and psychiatric conditions. While *ex vivo* studies using postmortem data have provided valuable insights into SWM organization,^42^^,^^43^ *in vivo* investigations remain limited. dMRI, as the only non-invasive tool for mapping white matter trajectories *in vivo*, has enabled substantial progress in the study of both DWM tracts and SWM pathways.^44^ DWM fibers, being longer, denser, and more stable, have been extensively characterized, leading to numerous atlases and segmentation frameworks.^21^^,^^45^^,^^46^ In contrast, SWM fibers are smaller, highly curved, located near the cortex, and exhibit greater inter-individual variability, making their reconstruction particularly challenging. Tractography errors in SWM are often driven by partial volume effects, complex fiber crossings,^2^ and variations in cortical folding,^22^ which can bias reconstructions and lead to underestimation of SWM pathways. These intrinsic anatomical features, combined with limitations of existing tractography methods, have hindered accurate and reproducible mapping of SWM *in vivo*.

To address these challenges, our study leveraged ultra-high-field Human Connectome Project (HCP) 7T data with multi-shell diffusion acquisition and multi-tissue constrained spherical deconvolution to generate fiber orientation distribution images with improved modeling of partial volume effects. The high spatial resolution of our dataset (1.05 mm isotropic) provided favorable conditions for delineating complex fiber trajectories, capturing local microstructural complexity, and improving SWM pathway reconstruction.^47^

Despite using a high-resolution dataset and extensive preprocessing to improve SWM pathway extraction, challenges such as false-positive fibers remain. To mitigate this, we applied outlier removal at both the individual and group levels, retaining all clusters that met the filtering criteria without further manual exclusion and providing uncertainty measures for each cluster. However, these procedures cannot fully exclude the possibility that some false-positive fibers remain. Therefore, the proposed atlas should be interpreted as a high-resolution, tractography-derived SWM resource with reproducible population-level organization, rather than as a definitive anatomical ground-truth atlas. The atlas also remains subject to false negatives introduced by the filtering procedure. Some plausible SWM clusters may have been excluded by the joint filtering, particularly if underrepresented in the training data or less statistically distinctive. Still, the large number of streamlines and redundancy across clusters suggested that a substantial proportion of plausible SWM pathways were retained. This flexible, cluster-based dataset may allow users to refine cluster selection according to their desired level of anatomical specificity in future studies.

Recently, several advanced methods have been proposed to improve the identification and reconstruction of SWM pathways. Surface-based tractography frameworks^24^^,^^48^^,^^49^^,^^50^ and deep learning approaches^31^ have shown great potential for enhancing the accuracy and automation of SWM mapping. However, their generalizability and robustness remain to be fully validated, and high-quality datasets derived from conventional methods continue to play a crucial role in SWM research.

### Limitations of the study

Several limitations should be considered when interpreting this resource. First, although the classifier was evaluated within the HCP 7T dataset, external validation of its generalization remains unavailable because comparable SWM/DWM labels were not available in the independent datasets. Therefore, the classifier results should be interpreted in the context of the labeled dataset used for atlas construction, rather than as evidence of fully validated cross-dataset generalization. Second, we did not perform a cluster-level split-half or test-retest reliability analysis. Future studies should further assess the stability of individual SWM clusters across repeated acquisitions, independent cohort partitions, and different tractography settings. Third, the cross-dataset TDI analysis supports reproducible SWM-like tractography patterns across high-quality dMRI datasets, but these results should not be interpreted as direct evidence of anatomical specificity. Finally, the TW-dFC-derived uncertainty metric and Neurosynth-based annotations were designed as descriptive aids for interpreting clusters, rather than as definitive evidence of functional organization or cognitive roles. These limitations should be taken into account when using the atlas as a tractography-derived resource or when adapting the proposed framework to new datasets.

## Resource availability

### Lead contact

Requests for further information and resources should be directed to and will be fulfilled by the lead contact, Ye Wu (wuye@njust.edu.cn).

### Materials availability

This study did not generate new unique reagents or biological materials. The tractography-derived superficial white matter atlas generated in this study is available as described in the data and code availability section.

### Data and code availability

• The tractography-derived SWM atlas generated in this study has been deposited and is publicly available at Science DataBank: https://doi.org/10.57760/sciencedb.28793. The SWM atlas was identified and extracted from a large-scale whole-brain fine-grained fiber cluster dataset resource,^51^ which has also been deposited and is publicly available at Science DataBank: https://doi.org/10.57760/sciencedb.28989. The SWM atlas resource includes SWM tractograms mapped to MNI152 space before and after hierarchical merging, cluster labels, and the correspondence between clusters under both the Yeo 7- and 17-network parcellation schemes. Data are provided in standard diffusion MRI and neuroimaging formats, including .tck and .nii.gz. Any future data updates related to this study will also be uploaded to the same Science Data Bank repository. Due to file storage limitations, subject-level fiber clusters before hierarchical merging are not included in the online resource but are available from the lead contact upon request. More
• Original code used for atlas generation, multi-criteria SWM pathway identification, hierarchical merging, and ICA analyses is available at GitHub: https://github.com/mushroomer1823/swm_atlas. The pipeline was implemented using MRtrix3, MATLAB, and in-house Python scripts and was tested in a high-performance computing environment.
• Any additional information required to reanalyze the data reported in this study is available from the lead contact upon request.

## Acknowledgments

This work was supported by the 10.13039/501100012166National Key R&D Program of China (No. 2023YFF1204803), the 10.13039/501100001809National Natural Science Foundation of China (No. 62201265), and the Key Project of Jiangsu Provincial Natural Science Fund (No. BK20253028).

## Author contributions

Conceptualization, Y. He and Y.W.; methodology, Y. He, Y. Hong, and Y.W.; investigation, Y. He, Y.X., and Y.W.; writing – original draft, Y. He, and H.Y.; writing – review and editing, Y. He, Y. Hong, and Y.W.; visualization: Y. He and Y.X.; funding acquisition, Y.W.; resources, Y.W.; supervision, Y. Hong and Y.W.

## Declaration of interests

The authors declare no competing interests.

## Declaration of generative AI and AI-assisted technologies in the writing process

During the preparation of this work, the authors used ChatGPT to improve the manuscript’s clarity and language and to generate selected visualization elements for the graphical abstract. After using this tool/service, the authors reviewed and edited the content as needed and take full responsibility for the article’s content.

## STAR★Methods

### Key resources table

**Table:** 

REAGENT or RESOURCE	SOURCE	IDENTIFIER
**Deposited data**		

Human Connectome Project 7T diffusion MRI data	Human Connectome Project	https://www.humanconnectome.org/study/hcp-young-adult/document/1200-subjects-data-release
Human Connectome Project 3T fMRI data	Human Connectome Project	https://www.humanconnectome.org/study/hcp-young-adult/document/extensively-processed-fmri-data-documentation
Detailed Connectomic Cluster Resource	Yip et al.	https://doi.org/10.57760/sciencedb.28989
Ultra-high-resolution diffusion MRI dataset	Wang et al.	https://doi.org/10.6084/m9.figshare.14058443
Ultra-high-gradient diffusion MRI dataset	Ramos-Llordén et al.	https://doi.org/10.1038/s41551-025-01457-x
Tractography-derived SWM atlas	This paper	https://doi.org/10.57760/sciencedb.28793

**Software and algorithms**		

Python	Python Software Foundation	https://www.python.org/
MRtrix3	Tournier et al.	https://www.mrtrix.org/
MATLAB	MathWorks	https://www.mathworks.com/products/matlab.html
Nibabel	NiBabel developers	https://nipy.org/nibabel/
Nilearn	Nilearn developers	https://nilearn.github.io
PyTorch	Paszke et al.	https://pytorch.org/
Scipy	Virtanen et al.	https://scipy.org/
Streamline to Cosine coefficients encoding code	This paper	https://github.com/mushroomer1823/connectomic_cluster
SWM atlas construction code	This paper	https://github.com/mushroomer1823/swm_atlas

**Other**		

Yeo 7- and 17-network parcellations	Yeo et al.	https://doi.org/10.1152/jn.00338.2011
FreeSurfer aparc.DKTatlas40 parcellation	FreeSurfer	https://surfer.nmr.mgh.harvard.edu/fswiki/CorticalParcellation
Neurosynth mata-analysis platform	Neurosynth	https://neurosynth.org

### Experimental model and study participant details

#### Human participants and diffusion MRI dataset

We utilized data from 171 healthy individuals collected using a 7.0T MRI scanner as part of the HCP.^52^ This dataset features high-resolution, high-quality, whole-brain dMRI data acquired using multiband acceleration. Specifically, T1-weighted and diffusion scans were obtained from the HCP subject release repository. The cohort comprised 104 female participants (60.82%) and 67 male participants (39.18%). Participants ranged in age from 22 to 36 years, with a mean age of 29.45 years. The race distribution was predominantly White (150 participants, 87.72%), followed by Black or African American (12 participants, 7.02%), Asian, Native Hawaiian, or other Pacific Islander (7 participants, 4.09%), and unknown or not reported race (2 participants, 1.17%). The original HCP data acquisition was approved by the Washington University Institutional Review Board (IRB #201204036), and written informed consent was obtained from all participants. Use of the HCP data in this study complied with the HCP Data Use Terms.

### Method details

#### Diffusion MRI dataset and preprocessing

All diffusion imaging data were acquired on a Siemens 7T MAGNETOM scanner equipped with an actively shielded system and a 32-channel head coil. Diffusion MRI was conducted using a spin-echo EPI sequence with a repetition time (TR) of 7,000 ms and an echo time (TE) of 71.2 ms. The flip angle was set to 90°, with a refocusing flip angle of 180°. The scans comprised 132 slices, each 1.05 mm thick, resulting in isotropic voxels of 1.05 mm in all directions. Imaging parameters also included a field of view (FOV) of 210 × 210 mm and a matrix size of 200 × 200. Acquisition efficiency was improved by using a 6/8-phase partial Fourier scheme, a multiband factor of 2, an in-plane acceleration factor (iPAT) of 3, and an echo spacing of 0.82 ms. Diffusion weighting was applied using b-values of 1000 and 2000 s/mm^2^, enabling sensitivity to varying degrees of diffusion. Prior studies have demonstrated that multi-shell dMRI, which incorporates multiple b-values, offers enhanced capability in resolving minor fiber populations compared to single-shell approaches. Furthermore, diffusion imaging at ultra-high field strength and higher spatial resolution provides a more detailed characterization of the FOD than the 3.0T HCP dataset. This level of detail is critical to identifying SWM, which is inherently more challenging to capture than DWM. All imaging datasets underwent the HCP Minimal Preprocessing Pipeline.^53^

All of the dMRI images were corrected for spatial distortions, imaging artifacts, and inter-modal misalignments. Rigid-body registration to the MNI152 standard space was performed using 6° of freedom. For dMRI data specifically, the pipeline standardized the b0 image intensity across sessions and applied corrections for EPI distortions, eddy currents, gradient nonlinearity, and subject motion. To generate FODs, a series of preprocessing and modeling steps was performed. A five-tissue-type (5 TT) segmentation file was derived from the cortical and subcortical parcellation. Tissue-specific response functions (RFs) were estimated for different b-values and tissue types by averaging diffusion-weighted signal profiles within each voxel, yielding RFs for white matter (WM), gray matter (GM), and cerebrospinal fluid (CSF). Multi-tissue fiber orientation distribution functions for WM, GM, and CSF were subsequently reconstructed. Based on these multi-tissue fODFs, both deterministic and probabilistic tractography were conducted.

#### Functional MRI dataset and preprocessing

For further functional analysis, functional MRI (fMRI) images were also acquired. Since the fMRI data from the HCP dataset primarily reflect gray matter signals, we used data from the HCP 3.0T dataset for the same set of subjects. All data were minimally preprocessed using the HCP pipeline^53^^,^^54^ and registered to the MNI152 standard space. Resting-state fMRI data were acquired using a gradient-echo EPI sequence with TR of 720 ms and TE of 33.1 ms, comprising 1200 time points across four runs, each lasting approximately 16 min. Resting-state fMRI data were further processed with a band-pass filter ranging from 0.01 to 0.1 Hz to retain relevant low-frequency fluctuations, which was conducted through the Nibabel Python toolbox https://pypi.org/project/nibabel/.

#### Construction of whole-brain fiber tractography

The pipeline for generating the whole-brain white matter fiber atlas is illustrated in Figure 1A. The overall pipeline first generated subject-level whole-brain tractograms, which were then aggregated at the population level and subsequently clustered into fine-grained fiber groups using k-means clustering. The resulting whole-brain fiber cluster dataset has been publicly released in,^51^ where more comprehensive details regarding its construction are provided.

To maximize the reconstruction of the genuine SWM pathway, two widely recognized probabilistic tractography algorithms, iFOD1 and iFOD2,^55^^,^^56^ along with a deterministic tractography algorithm SD_STREAM,^55^ were implemented separately to generate the initial tractography for each subject. The three tracking algorithms were applied with different combinations of seeding strategies, angular constraints, and step sizes. See Table 2 for details. 4th-order Runge-Kutta (RK4) integration was applied to eliminate curvature overshoot in 1st-order deterministic methods, and cropping at the gray-white matter interface was enabled. Other default parameters followed MRtrix3 defaults for HCP-like datasets: a FOD cutoff of 0.01 and a streamline length constrained between 20 mm and 250 mm, with Anatomically Constrained Tractography (ACT)^57^ enabled and the crop streamline endpoints set to be more precise as they cross the GM-WM interface. The resulting tractograms were merged to create a final whole-brain tractogram at the population level.

All three fiber tracking algorithms above have been widely employed for tractography reconstruction in previous studies. However, no tractography method to date has performed flawlessly in terms of both sensitivity and specificity. From a general perspective, deterministic methods offer high specificity. Still, they may miss complex or ambiguous pathways, whereas probabilistic methods better capture uncertainty and crossing fibers, albeit at the cost of increased false positives and lower anatomical precision. When it comes to SWM, deterministic and probabilistic tractography showed different reproducibility and sensitivity in reconstructing SWM pathway bundles.^9^^,^^23^^,^^58^ In this study, we aimed to achieve the most comprehensive and robust extraction of the SWM pathway by integrating multiple tractography methods, thereby enabling a more complete and reliable mapping of SWM architecture. The concept of ensemble tractography inspires this strategy,^59^^,^^60^^,^^61^ aiming to increase sensitivity and reduce algorithm-specific dependency by combining multiple tracking methods, rather than claiming a definitively superior reconstruction approach. To further support this design choice, we performed a focused ablation analysis on a high-quality ultra-strong-gradient diffusion MRI sample,^13^ comparing SWM-related track density distributions generated by different tractography algorithms and parameter settings. The results, reported in the Supplementary Material (Figures S1 and S2), showed method-dependent differences in local track density patterns, supporting the use of multiple complementary tracking strategies. After tractography reconstruction, two outlier-removal and false-positive-elimination steps were performed at the individual and population levels.^62^^,^^63^ For each subject, the tractograms generated by the three tracking algorithms under all parameter settings were then concatenated using the tckedit command in MRtrix3 to form a single whole-brain tractogram, which was subsequently used for population-level tractogram construction.

Cortical parcellation was based on the Yeo functional atlas,^36^ which delineates functional regions using data-driven clustering of resting-state fMRI data, thereby reducing biases relative to anatomical parcellations and improving functional connectivity analyses. Subcortical regions were segmented into nine areas, including the cerebellum, thalamus, basal ganglia, hippocampus, amygdala, accumbens, and brainstem, enabling precise assessment of cortical–subcortical interactions. The Yeo atlas provides 7- and 17-network schemes, and our SWM extraction pipeline was applied to both, generating two corresponding SWM atlas versions for subsequent analyses.

#### Tractography ablation analysis

To assess the influence of tractography algorithms and parameter settings on reconstructed fiber distributions, we performed a focused ablation analysis using an ultra-high-gradient *in vivo* human diffusion MRI dataset. Whole-brain tractograms were reconstructed using SD_STREAM, iFOD1, and iFOD2 under the same set of parameter combinations used for the main tractography reconstruction. For each tractogram, TDIs were generated using the tckmap command in MRtrix3 and min–max normalized. Normalized TDIs were converted into binary masks using thresholds of 0, 0.25, 0.5, and 0.75, and pairwise Dice coefficients were computed between masks generated from different tractography algorithms and parameter settings. For visualization, mean normalized TDI maps were computed for each tractography algorithm by averaging across all corresponding parameter settings. The resulting Dice overlap matrices and mean-normalized TDI maps are shown in Figures S1 and S2.

#### Quantity-adaptive group-wise fiber clustering

To prepare for the subsequent clustering step, we first extracted a feature representation of each whole-brain fiber streamline, known as the cosine coefficient representation,^64^ which captures both its shape and spatial location. Rather than using the original streamline coordinates, which are memory-intensive, the cosine coefficient representation is more compact and representative, which has been proven effective and valid for fiber distinction,^62^^,^^65^^,^^66^ free of streamline resampling. First, the coordinate matrix of each streamline, with a shape of *n* × 3, was extracted, where *n* is the number of points. Then, the original coordinate matrix was transformed into a cosine-coefficient matrix with shape 5 × 3, with the first row describing the streamline’s position. At the same time, the other four rows represented its shape. In the subsequent clustering steps, the cosine coefficient matrix was flattened into a 12-dimensional vector, representing each streamline’s features.

To obtain more structurally refined fiber subtypes, we clustered the fiber streamlines connecting 7- or 17-networks and 9 subcortical structures. Specifically, fiber streamlines were classified into distinct categories based on the region of interest (ROI) pair to which they were connected. The fiber streamlines within each ROI pair were clustered separately.

Streamlines were represented using cosine coefficients and clustered with k-means to improve robustness. Given the high-dimensional, manifold structure of fibers, traditional heuristic rules are suboptimal; we empirically set the number of clusters to $\left\lfloor \sqrt[{5}]{n}\right\rfloor$, where *n* is the number of streamlines, ensuring comparable cluster sizes and meaningful grouping (Figure 3A).

#### Superficial white matter fiber identification

The overall pipeline for constructing the whole-brain atlas and selecting the SWM is illustrated in Figure 1B. To separate the SWM from the whole-brain tractogram, SWM clusters were first filtered based on their shape, location, and length. To identify as many real SWM pathways as possible, we adopted relatively lenient criteria for each specific feature (e.g., length, curvature). However, by applying multiple criteria, fibers that did not belong to the SWM were progressively excluded. The specific thresholds were set based on references to morphological studies of the SWM pathway.^8^^,^^9^^,^^47^^,^^67^ After careful consideration, the SWM pathway must meet the following criteria:

(Equation 1) $$\begin{array}{c} 15mm<l<85mm, \end{array}$$

(Equation 2) $$\begin{array}{c} r_{\mathrm{mid-length}}<2,\;r_{\mathrm{mid-length}}=\frac{d_{\mathrm{mid}}}{l}, \end{array}$$

(Equation 3) $$\begin{array}{c} r_{\mathrm{mid-end}}>1.1,\;r_{\mathrm{mid-end}}=\frac{d_{\mathrm{mid}}}{d_{\mathrm{end}}}, \end{array}$$

(Equation 4) $$\begin{array}{c} r_{\mathrm{length-end}}>1.1,\;r_{\mathrm{length-end}}=\frac{l}{d_{\mathrm{end}}}, \end{array}$$

(Equation 5) $$\begin{array}{c} r_{cc}<0.01,\;r_{cc}=\frac{\min\left(R,L\right)}{\max\left(R,L\right)} \end{array}$$

where *l* is the length of the fiber, *d*_*end*_ is the Euclidean distance between the two endpoints of the fiber, and *d*_*mid*_ is the sum of the distance from the fiber’s starting point to its midpoint and that from the midpoint to its endpoint. *R* and *L* represent the number of points on the fiber streamline in the right or left hemisphere, respectively. *r*_*mid−length*_, *r*_*mid−end*_, and *r*_*length−end*_ were designed to constrain the curvature of the fiber streamlines.

Each fiber cluster was assigned to a probability value $P_{\mathrm{geometric}}^{k}$ representing the likelihood of the cluster *k* belonging to SWM from the geometric perspective. The specific calculation method is as follows:

(Equation 6) $$P_{\mathrm{geometric}}^{k}=\frac{n_{SWM\;geometric\;\mathrm{fi}bers}}{n_{\mathrm{fibers}}^{k}}$$

where *n*_*SWM*_ _*geometric*_ _*fibers*_ is the number of fiber streamlines satisfying the geometric criteria of being SWM, and $n_{\mathrm{fibers}}^{k}$ is the total number of fiber streamlines in the fiber cluster *k*.

In addition, due to the property of the SWM pathway connecting only adjacent brain regions, we incorporated both the Desikan-Killiany atlas, also known as the DKT atlas,^68^ and the Yeo atlas, as references to determine the adjacency of brain regions on top of the network parcellation, thereby further filtering out fibers connecting non-adjacent regions. Specifically, a fiber was considered an SWM fiber only if it connected regions that were adjacent in both the DKT and Yeo atlases. This dual-atlas constraint provides a more stringent criterion, allowing us to exclude additional fibers that link non-adjacent regions and improve the specificity of SWM identification. The adjacency relationships between ROIs in both atlases were computed automatically based on spatial contiguity. The cortical parcellations of the Yeo and DKT atlases, together with the corresponding ROI adjacency heatmaps, are visualized in Figure S5.

In addition to position- and geometry-based filters, we developed a neural network classifier to automatically determine the probability that a fiber cluster belongs to the SWM. The training dataset from the ORG atlas^21^ included hundreds of fiber bundles labeled as SWM or non-SWM. In the ORG atlas, SWM clusters are initially identified based on the anatomical regions traversed by fibers, with clusters involving deep white matter structures first excluded. The remaining clusters are retained if they connect the same or adjacent brain lobes (e.g., frontal and parietal lobes), and the final annotations are further refined through expert neuroanatomical validation. We first used asymmetric fiber orientation distribution functions to represent the shape and position features of each fiber.^69^ Each fiber streamline was transformed into a 5 × 3 cosine coefficient matrix. The first row captures the streamline’s spatial position, while the remaining four rows encode its shape characteristics using higher-order cosine terms. This compact representation, referred to as the cosine coefficient representation, is both efficient and effective for distinguishing between streamline trajectories.^65^ We employed a simple multilayer perceptron (MLP) as a baseline classifier to distinguish SWM fibers from non-SWM fibers. The MLP consisted of four fully connected layers with hidden dimensions of 128, 256, and 64, respectively. ReLU activations were applied after each hidden layer. The network was trained using the cross-entropy loss function. Optimization was performed using the Adam optimizer with an initial learning rate of 1 × 10^−3^. Training was conducted for 10 epochs with a batch size of 128. To accelerate training, data-parallel learning was employed on two RTX3090 GPUs using PyTorch’s DataParallel. Model performance was evaluated on independent validation and test sets using classification accuracy and loss. A test set consisting of 15% of the fibers from the ORG atlas that were not used for training was used for evaluation, yielding an accuracy of 0.95, a precision of 0.89, a recall of 0.92, and an F1-score of 0.90.

The trained neural network outputs a binary classification (true or false) for the encoded SWM feature, indicating whether a fiber belongs to the SWM. The probability $P_{\mathrm{network}}^{k}$ of the fiber cluster *k* belonging to SWM from the perspective of the neural network was calculated as:

(Equation 7) $$P_{\mathrm{network}}^{k}=\frac{n_{SWM\;network\;\mathrm{fi}bers}^{k}}{n_{\mathrm{fibers}}^{k}}$$

where $n_{SWM\;network\;\mathrm{fi}bers}^{k}$ is the number of fiber streamlines determined as SWM by the neural network classifier in the fiber cluster *k*, and $n_{\mathrm{fibers}}^{k}$ is the total number of fiber streamlines in the fiber cluster *k*.

All fiber clusters were assigned SWM probabilities using geometric feature-based filtering and a neural network classifier. We then computed the joint probability by multiplying the two probability values. Next, we calculated Z-scores for the SWM probabilities from the three approaches across all fiber clusters and selected fiber clusters in which the Z-scores for all three probabilities exceeded 1.5 as the identified SWM clusters. The standard *Z* score was calculated using the *Z* score function in the SciPy Python library. Under the 7-network parcellation, geometric feature-based filtering alone retained 4,758 candidate clusters. After incorporating the deep learning-based network classifier, 2,680 of these candidates were further removed, indicating that both classifiers played important and complementary roles in the filtering process. Compared with geometric filtering alone, the network classifier excluded a substantial proportion of candidate clusters that met the shape- and position-based criteria yet exhibited connectivity patterns inconsistent with SWM definitions. To further clarify the classifier’s contribution, we now provide representative examples in Figure S6, showing clusters that were retained by the geometric criteria but excluded by the network classifier. These examples illustrate that the classifier primarily removed geometrically plausible fibers whose connectivity patterns were inconsistent with the expected characteristics of SWM. Finally, because SWM only connects adjacent brain regions, we refined the identified clusters by removing those that did not meet this adjacency criterion.

Additional cross-subject consistency analysis was conducted to assess whether the selected SWM pathways could be reconstructed in individual subject tractograms. This analysis supports the population-level reproducibility of the identified tractography patterns. To quantify the validation results, we calculated the consistency metric *C*_*k*_ as follows:

(Equation 8) $$C^{k}=\frac{n_{valid\;subjects}^{k}}{n_{\mathrm{subjects}}}\times 100\%$$

where *n*_*valid*_ _*subjects*_ represents the number of subjects in whose whole-brain tractograms the target cluster *k* was successfully reconstructed (defined as having at least one fiber in the corresponding cluster), while *n*_*subjects*_ indicates the total number of subjects included in the analysis.

#### Hierarchical clustering to merge similar SWM clusters

To prevent the generation of numerous SWM clusters that lack meaningful distinctions, hierarchical clustering^70^ was used to merge clusters with similar characteristics. Hierarchical clustering is an unsupervised learning algorithm that builds a nested hierarchy of clusters by successively merging or splitting them based on similarity (see the schematic diagram in Figure 3B). It does not require a predefined number of clusters and produces a dendrogram that reflects the relationships among data points at different levels of granularity. In this study, Hierarchical clustering was performed using the Hierarchy module from the SciPy library https://scipy.org/.

For each SWM cluster, we initially calculated an average fiber streamline by taking the mean cosine coefficient vector of all streamlines in that cluster. We then employed hierarchical clustering on these average cosine coefficients to assess pairwise similarities, with smaller Euclidean distances between clusters indicating greater similarity. Cluster merging was performed using a series of predefined distance thresholds, ranging from 0.5 to 50.0 in increments of 0.5. Clusters with a distance below the selected threshold were combined. Notably, hierarchical clustering was applied at the connection-pair level, considering only those clusters that connected the same pair of functional networks or subcortical regions for integration.

To evaluate clustering performance, we employed the Davies–Bouldin index^71^ and determined the optimal merging threshold using the elbow point method.^72^ The elbow point method is a heuristic for determining the optimal number of clusters by identifying the point at which the rate of improvement in a clustering metric sharply decreases (Figure 3C). This approach strikes a balance between model complexity and clustering quality, thereby avoiding overfitting or under-segmentation, and has been widely employed in related research.^73^

#### Construction of TW-dFC images for structural and functional information integration

Each fiber streamline in the SWM tractogram was assigned a weight by calculating the Pearson functional correlation index of the endpoints in the cortex. Track endpoints were defined using ACT.^57^ The functional weight of each voxel was then defined as the average of all streamlined weights that passed through it. It should be noted that the voxel weights were dynamic (time-dependent), rather than static, implemented using a sliding Hamming time window of 40 s (55 volumes, TR = 0.72 s). The TW-dFC value for the voxel at the time point can be calculated as:

(Equation 9) $$TWdFC{\left(v,t\right)}_{\mathrm{SWM}}=\frac{1}{N_{v}}\overset{N_{v}}{\underset{i=1}{\sum }}FC_{i}\left(t\right),$$

where *N*_*v*_ is the number of SWM streamlines traversing the voxel *v*, and *FC* is the Pearson correlation index of the functional signals on the streamline’s endpoints. In this work, we used resting-state and 7-task fMRI images as independent inputs, yielding 8 TW-dFC images per subject.

#### Independent components analysis for voxel-level parcellation

To obtain a more structurally and functionally consistent parcellation of SWM voxels, we performed ICA on the TW-dFC images. Compared with conventional resting-state-based ICA, this approach simultaneously incorporates both structural and functional information. After representing the SWM with a fiber-weighted functional connectivity image using TW-dFC, we performed CanICA^74^ from the Nilearn library to separate the group-level independent sources and estimate the mixing model. Group-level spatial ICA is a widely used methodology for analyzing fMRI data by decomposing fMRI data into ICs, where each component represents a distinct spatial map of brain activity.^75^ These ICs correspond to neural networks or functional brain regions that are temporally coherent yet spatially independent, exhibiting distinct activity patterns. The ICA results provided 3D mapping for each IC, highlighting the voxels correlated with the specific IC pattern, with positive or negative weights. We extracted the weight vector of each voxel by concatenating the weight values as a feature embedding. Each voxel was assigned to the corresponding independent component category based on the feature dimension with the largest value in its feature vector.

There is no clear standard for selecting the number of independent components.^76^ In previous studies that implemented ICA, the number of independent components is typically set between 10 and 250, often determined empirically or based on spatial correlations.^77^^,^^78^^,^^79^ Studies utilizing whole-brain resting-state fMRI data commonly select a value around 20.^80^ However, given that our data consist of TW-dFC signals confined to SWM voxels and that we used a combined representation of eight functional modalities, we believe that empirically determined IC numbers may not be directly applicable to our dataset. To automatically determine the optimal number of ICs, a series of IC numbers (ranging from 40 to 120 in increments of 5) was used to implement ICA on TW-dFC images across all 8 tasks. DB indices were calculated to quantify the final clustering performance. For the resting-state data, 80 was chosen as the number of ICs based on a relatively low DB index for both the 7- and 17-parcel schemes, upon which ICA was subsequently conducted. A similar procedure was applied to determine the number of ICs for other task-based fMRI conditions. The process of selecting the optimal number of ICs is illustrated in Figure 5D. When the IC numbers corresponding to the minimum DB index differ between the 7- and 17-network schemes, we select the IC number corresponding to the lowest point of the fitted DB index curve.

#### Definition of SWM cluster uncertainty under strict and relaxed IC rules

Based on the IC parcellation of the SWM at the voxel level, each fiber streamline can be represented by an embedding of the corresponding voxel labels. Each streamline was resampled to 30 points, and a corresponding category label was assigned based on the voxel in which it was located. The structural-functional coherence of the SWM cluster was measured by the probability that its points were assigned to similar components (either the same component or the two most probable ones). If points within a cluster appear in multiple functional partitions, the cluster is considered highly uncertain. The uncertainty value is defined as

(Equation 10) $$P_{\mathrm{similarity}}^{k}=\frac{N_{same\;M\;ICs}^{k}}{N_{\mathrm{points}}^{k}},\;M\in \left\{1,2\right\}$$

(Equation 11) $$P_{\mathrm{uncertainty}}^{k}=1-P_{\mathrm{similarity}}^{k}$$

where $P_{\mathrm{uncertainty}}^{k}$ is the uncertainty metric of the SWM cluster *k*. $P_{\mathrm{similarity}}^{k}$ is the probability that points in the SWM cluster *k* belong to one or two of the same components, as $N_{\mathrm{points}}^{k}$ represents the total number of points in the SWM cluster *k*. $N_{same\;M\;ICs}^{k}$ is the number of fiber points in the same *M* ICs for cluster *k*. *M* = 1 or 2 corresponds to the strict and relaxed IC rule, respectively.

### Quantification and statistical analysis

All quantitative analyses were performed at the subject, streamline, cluster, or voxel level as specified in the corresponding results and STAR Methods sections and figure legends. The primary cohort included 171 HCP participants. Cross-subject consistency was calculated across these 171 participants. Cluster-level analyses used *n* = 643 clusters for the Yeo 7-network atlas and *n* = 1,403 clusters for the Yeo 17-network atlas after hierarchical merging. TDI similarity analyses were performed using SSIM, LPIPS, and PSNR to compare atlas-derived SWM TDIs with TDIs generated from independent high-quality diffusion MRI datasets. For regression analyses of TW-dFC-derived uncertainty, statistical significance was defined as *p* < 0.001. Data processing and statistical analyses were performed using Python, MATLAB, MRtrix3, SciPy, Nilearn, and related neuroimaging tools as described in the method details.

#### Control analysis of structural factors associated with TW-dFC-derived uncertainty

Regression analyses were used to assess whether the strict and relaxed TW-dFC-derived uncertainty metrics were associated with basic structural properties of SWM clusters. Three cluster-level structural variables were included: fiber count, spatial span, and mean streamline length. For each uncertainty metric, we first fitted separate univariable linear regression models using each structural variable as the explanatory variable. We then fitted a multivariable linear regression model including all three structural variables jointly. Model fit was summarized using the coefficient of determination (*R*^2^), which represents the proportion of variance in the uncertainty metric explained by the corresponding structural variable or by the combined multivariable model. Statistical significance of the regression models was assessed using a threshold of *p* < 0.001. The resulting univariable and multivariable regression results are summarized in Figure S3 and Table S1.

## References

[1] Schüz A., Braitenberg V., Schüz A., Miller R. (2002). Cortical areas.

[2] Guevara M., Guevara P., Román C., Mangin J.F. (2020). Superficial white matter: A review on the dmri analysis methods and applications. Neuroimage.

[3] Kirilina E., Helbling S., Morawski M., Pine K., Reimann K., Jankuhn S., Dinse J., Deistung A., Reichenbach J.R., Trampel R. (2020). Superficial white matter imaging: Contrast mechanisms and whole-brain in vivo mapping. Sci. Adv..

[4] Schilling K.G., Archer D., Rheault F., Lyu I., Huo Y., Cai L.Y., Bunge S.A., Weiner K.S., Gore J.C., Anderson A.W., Landman B.A. (2023). Superficial white matter across development, young adulthood, and aging: volume, thickness, and relationship with cortical features. Brain Struct. Funct..

[5] Pietrasik W., Cribben I., Olsen F., Malykhin N. (2023). Diffusion tensor imaging of superficial prefrontal white matter in healthy aging. Brain Res..

[6] Schilling K.G., Archer D., Yeh F.C., Rheault F., Cai L.Y., Shafer A., Resnick S.M., Hohman T., Jefferson A., Anderson A.W. (2023). Short superficial white matter and aging: a longitudinal multi-site study of 1293 subjects and 2711 sessions. Aging Brain.

[7] Wang S., Zhang F., Huang P., Hong H., Jiaerken Y., Yu X., Zhang R., Zeng Q., Zhang Y., Kikinis R. (2022). Superficial white matter microstructure affects processing speed in cerebral small vessel disease. Hum. Brain Mapp..

[8] Van Dyken P.C., Khan A.R., Palaniyappan L. (2024). Imaging of the superficial white matter in health and disease. Imaging Neurosci..

[9] Guevara M., Román C., Houenou J., Duclap D., Poupon C., Mangin J.F., Guevara P. (2017). Reproducibility of superficial white matter tracts using diffusion-weighted imaging tractography. Neuroimage.

[10] Veale T., Malone I.B., Poole T., Parker T.D., Slattery C.F., Paterson R.W., Foulkes A.J.M., Thomas D.L., Schott J.M., Zhang H. (2021). Loss and dispersion of superficial white matter in alzheimer’s disease: a diffusion mri study. Brain Commun..

[11] Ming Z., He Y., Xie Y., Ni H., Yao Z., Lu Q., Wu Y., Qin J. (2026). 2026 IEEE 23rd International Symposium on Biomedical Imaging (ISBI).

[12] Wang F., Dong Z., Tian Q., Liao C., Fan Q., Hoge W.S., Keil B., Polimeni J.R., Wald L.L., Huang S.Y., Setsompop K. (2021). In vivo human whole-brain connectom diffusion mri dataset at 760 *μ*m isotropic resolution. Sci. Data.

[13] Ramos-Llordén G., Lee H.H., Davids M., Dietz P., Krug A., Kirsch J.E., Mahmutovic M., Müller A., Ma Y., Lee H. (2026). Ultra-high gradient connectomics and microstructure mri scanner for imaging of human brain circuits across scales. Nat. Biomed. Eng..

[14] Wang Z., Bovik A.C., Sheikh H.R., Simoncelli E.P. (2004). Image quality assessment: from error visibility to structural similarity. IEEE Trans. Image Process..

[15] Zhang R., Isola P., Efros A.A., Shechtman E., Wang O. (2018). Proceedings of the IEEE conference on computer vision and pattern recognition.

[16] Huynh-Thu Q., Ghanbari M. (2008). Scope of validity of psnr in image/video quality assessment. Electron. Lett..

[17] Oishi K., Zilles K., Amunts K., Faria A., Jiang H., Li X., Akhter K., Hua K., Woods R., Toga A.W. (2008). Human brain white matter atlas: identification and assignment of common anatomical structures in superficial white matter. Neuroimage.

[18] Guevara P., Duclap D., Poupon C., Marrakchi-Kacem L., Fillard P., Le Bihan D., Leboyer M., Houenou J., Mangin J.F. (2012). Automatic fiber bundle segmentation in massive tractography datasets using a multi-subject bundle atlas. Neuroimage.

[19] Guevara M., Román C., Houenou J., Duclap D., Poupon C., Mangin J.F., Guevara P. (2016). 2016 38th Annual International Conference of the IEEE Engineering in Medicine and Biology Society (EMBC).

[20] Román C., Guevara M., Valenzuela R., Figueroa M., Houenou J., Duclap D., Poupon C., Mangin J.F., Guevara P. (2017). Clustering of Whole-Brain White Matter Short Association Bundles Using HARDI Data. Front. Neuroinform..

[21] Zhang F., Wu Y., Norton I., Rigolo L., Rathi Y., Makris N., O’Donnell L.J. (2018). An anatomically curated fiber clustering white matter atlas for consistent white matter tract parcellation across the lifespan. Neuroimage.

[22] Guevara M., Sun Z.Y., Guevara P., Rivière D., Grigis A., Poupon C., Mangin J.F. (2022). Disentangling the variability of the superficial white matter organization using regional-tractogram-based population stratification. Neuroimage.

[23] Román C., Hernández C., Figueroa M., Houenou J., Poupon C., Mangin J.F., Guevara P. (2022). Superficial white matter bundle atlas based on hierarchical fiber clustering over probabilistic tractography data. Neuroimage.

[24] Li Y., Nie X., Zhang J., Shi Y., Linguraru M.G., Dou Q., Feragen A., Giannarou S., Glocker B., Lekadir K., Schnabel J.A. (2024). International Conference on Medical Image Computing and Computer-Assisted Intervention.

[25] Roebroeck A., Galuske R., Formisano E., Chiry O., Bratzke H., Ronen I., Kim D.s., Goebel R. (2008). High-resolution diffusion tensor imaging and tractography of the human optic chiasm at 9.4 t. Neuroimage.

[26] Gulban O.F., De Martino F., Vu A.T., Yacoub E., Uğurbil K., Lenglet C. (2018). Cortical fibers orientation mapping using in-vivo whole brain 7 t diffusion mri. Neuroimage.

[27] Obusez E.C., Lowe M., Oh S.H., Wang I., Bullen J., Ruggieri P., Hill V., Lockwood D., Emch T., Moon D. (2018). 7t mr of intracranial pathology: preliminary observations and comparisons to 3t and 1.5 t. Neuroimage.

[28] Nazeri A., Chakravarty M.M., Rajji T.K., Felsky D., Rotenberg D.J., Mason M., Xu L.N., Lobaugh N.J., Mulsant B.H., Voineskos A.N. (2015). Superficial white matter as a novel substrate of age-related cognitive decline. Neurobiol. Aging.

[29] d’Albis M.A., Guevara P., Guevara M., Laidi C., Boisgontier J., Sarrazin S., Duclap D., Delorme R., Bolognani F., Czech C. (2018). Local structural connectivity is associated with social cognition in autism spectrum disorder. Brain.

[30] Hatton S.N., Lagopoulos J., Hermens D.F., Hickie I.B., Scott E., Bennett M.R. (2014). Short association fibres of the insula-temporoparietal junction in early psychosis: a diffusion tensor imaging study. PLoS One.

[31] Xue T., Zhang F., Zhang C., Chen Y., Song Y., Golby A.J., Makris N., Rathi Y., Cai W., O’Donnell L.J. (2023). Superficial white matter analysis: An efficient point-cloud-based deep learning framework with supervised contrastive learning for consistent tractography parcellation across populations and dmri acquisitions. Med. Image Anal..

[32] Wang T., Wan Z., Cao S., Yu J., He Y., Xie Y., Zhang F., Wu Y. (2026). Deep learning empowered microstructure codebook: New paradigm for multi-parameter tissue characterization estimation. Hum. Brain Mapp..

[33] Zhai Y., He Y., Wan Z., Xie Y., Qin J., Wu Y., Liu J., Huang J., Wang X., Zhang F., Zou Z., Tian T., Hu X., Hu B., Xiong Y. (2025). 2025 IEEE International Conference on Bioinformatics and Biomedicine (BIBM).

[34] Bigham B., Zamanpour S.A., Zemorshidi F., Boroumand F., Zare H., Alzheimer’s Disease Neuroimaging Initiative (2020). Identification of superficial white matter abnormalities in alzheimer’s disease and mild cognitive impairment using diffusion tensor imaging. J. Alzheimers Dis. Rep..

[35] Liu M., Bernhardt B.C., Hong S.J., Caldairou B., Bernasconi A., Bernasconi N. (2016). The superficial white matter in temporal lobe epilepsy: a key link between structural and functional network disruptions. Brain.

[36] Yeo B.T., Krienen F.M., Sepulcre J., Sabuncu M.R., Lashkari D., Hollinshead M., Roffman J.L., Smoller J.W., Zöllei L., Polimeni J.R. (2011). The organization of the human cerebral cortex estimated by intrinsic functional connectivity. J. Neurophysiol..

[37] Fotiadis P., Parkes L., Davis K.A., Satterthwaite T.D., Shinohara R.T., Bassett D.S. (2024). Structure–function coupling in macroscale human brain networks. Nat. Rev. Neurosci..

[38] Popp J.L., Thiele J.A., Faskowitz J., Seguin C., Sporns O., Hilger K. (2024). Structural-functional brain network coupling predicts human cognitive ability. Neuroimage.

[39] Zarkali A., McColgan P., Leyland L.A., Lees A.J., Rees G., Weil R.S. (2021). Organisational and neuromodulatory underpinnings of structural-functional connectivity decoupling in patients with parkinson’s disease. Commun. Biol..

[40] Ruthig P., von der Planitz D.E., Morozova M., Reimann K., Jäger C., Reinert T., Mohammadi S., Weiskopf N., Kirilina E., Morawski M. (2025). Short-range human cortico-cortical white matter fibers have thinner axons and are less myelinated compared to long-range fibers despite a similar g-ratio. PLoS Biol..

[41] Peer M., Nitzan M., Bick A.S., Levin N., Arzy S. (2017). Evidence for functional networks within the human brain’s white matter. J. Neurosci..

[42] Wilkinson M., Wang R., van der Kouwe A., Takahashi E. (2016). White and gray matter fiber pathways in autism spectrum disorder revealed by ex vivo diffusion mr tractography. Brain Behav..

[43] Reveley C., Seth A.K., Pierpaoli C., Silva A.C., Yu D., Saunders R.C., Leopold D.A., Ye F.Q. (2015). Superficial white matter fiber systems impede detection of long-range cortical connections in diffusion MR tractography. Proc. Natl. Acad. Sci. USA.

[44] Basser P.J., Pajevic S., Pierpaoli C., Duda J., Aldroubi A. (2000). In vivo fiber tractography using dt-mri data. Magn. Reson. Med..

[45] Li Y., Zhang W., Wu Y., Yin L., Zhu C., Chen Y., Cetin-Karayumak S., Cho K.I.K., Zekelman L.R., Rushmore J. (2024). A diffusion mri tractography atlas for concurrent white matter mapping across eastern and western populations. Sci. Data.

[46] Adil S.M., Calabrese E., Charalambous L.T., Cook J.J., Rahimpour S., Atik A.F., Cofer G.P., Parente B.A., Johnson G.A., Lad S.P., White L.E. (2021). A high-resolution interactive atlas of the human brainstem using magnetic resonance imaging. Neuroimage.

[47] Zhang F., Chen Y., Ning L., Rushmore J., Liu Q., Du M., Hassanzadeh-Behbahani S., Legarreta J.H., Yeterian E., Makris N. (2024). Assessment of the depiction of superficial white matter using ultra-high-resolution diffusion mri. Hum. Brain Mapp..

[48] Gahm J.K., Shi Y., Shen D., Liu T., Peters T.M., Staib L.H., Essert C., Zhou S., Yap P.-T., Khan A. (2019).

[49] Wu Y., Hong Y., Ahmad S., Yap P.T., de Bruijne M., Cattin P.C., Cotin S., Padoy N., Speidel S., Zheng Y., Essert C.. (2021). International Conference on Medical Image Computing and Computer-Assisted Intervention.

[50] Nie X., Ruan J., Otaduy M.C.G., Grinberg L.T., Ringman J., Shi Y. (2024). Surface-based probabilistic fiber tracking in superficial white matter. IEEE Trans. Med. Imaging.

[51] Yip H., He Y., Xie Y., Zhang F., Wu Y. (2026). Detailed Connectomic Cluster Resource for White Matter Mapping From Ultra-High-Field Diffusion MRI. Neuroimage.

[52] Vu A.T., Auerbach E., Lenglet C., Moeller S., Sotiropoulos S.N., Jbabdi S., Andersson J., Yacoub E., Ugurbil K. (2015). High resolution whole brain diffusion imaging at 7 t for the human connectome project. Neuroimage.

[53] Glasser M.F., Sotiropoulos S.N., Wilson J.A., Coalson T.S., Fischl B., Andersson J.L., Xu J., Jbabdi S., Webster M., Polimeni J.R. (2013). The minimal preprocessing pipelines for the human connectome project. Neuroimage.

[54] Smith S.M., Beckmann C.F., Andersson J., Auerbach E.J., Bijsterbosch J., Douaud G., Duff E., Feinberg D.A., Griffanti L., Harms M.P. (2013). Resting-state fmri in the human connectome project. Neuroimage.

[55] Tournier J.D., Calamante F., Connelly A. (2012). Mrtrix: diffusion tractography in crossing fiber regions. Int. J. Imaging Syst. Technol..

[56] Tournier J.D., Calamante F., Connelly A. (2010).

[57] Smith R.E., Tournier J.D., Calamante F., Connelly A. (2012). Anatomically-constrained tractography: improved diffusion mri streamlines tractography through effective use of anatomical information. Neuroimage.

[58] Schilling K., Zhang F., Román C., O’Donnell L.J., Guevara P. (2025). Short association fiber tractography: key insights and surprising facts. Brain Struct. Funct..

[59] Takemura H., Caiafa C.F., Wandell B.A., Pestilli F. (2016). Ensemble tractography. PLoS Comput. Biol..

[60] Li S., Zhang W., Yao S., He J., Gao J., Xue T., Xie G., Chen Y., Torio E.F., Feng Y. (2024). Tractography-based automated identification of retinogeniculate visual pathway with novel microstructure-informed supervised contrastive learning. Hum. Brain Mapp..

[61] Wan Z., Wang P., Zhai Y., Xie Y., He Y., Wu Y. (2026). Constructing fine-grained subcortical atlases with connectional consensus graph representation learning. Phys. Med. Biol..

[62] Wu Y., Ahmad S., Yap P.T., de Bruijne M., Cattin P.C., Cotin S., Padoy N., Speidel S., Zheng Y., Essert C. (2021). International Conference on Medical Image Computing and Computer-Assisted Intervention.

[63] Yeh F.C., Panesar S., Barrios J., Fernandes D., Abhinav K., Meola A., Fernandez-Miranda J.C. (2019). Automatic removal of false connections in diffusion mri tractography using topology-informed pruning (tip). Neurotherapeutics.

[64] Adluru N., Alexander A.L., Chung M.K., Lainhart J.E., Lazar M., Lee J.E. (2010). Cosine series representation of 3d curves and its application to white matter fiber bundles in diffusion tensor imaging. Stat. Interface.

[65] Wu Y., Hong Y., Ahmad S., Lin W., Shen D., Yap P.T., Consortium U.B.C.P., Martel A.L., Abolmaesumi P., Stoyanov D., Mateus D., Zuluaga M.A., Zhou S.K., Racoceanu D., Joskowicz L. (2020). International Conference on Medical Image Computing and Computer-Assisted Intervention.

[66] Xie Y., Zhai Y., He Y., Yu J., Qin J., Wu Y., Cannataro M., Zheng H.J., Gao L., Cheng J., Luís de Miranda J., Zumpano E., Hu X., Cho Y.-R., Park T. (2024). 2024 IEEE International Conference on Bioinformatics and Biomedicine (BIBM).

[67] Makris N., Rushmore R., Kaiser J., Albaugh M., Kubicki M., Rathi Y., Zhang F., O’Donnell L.J., Yeterian E., Caviness V.S., Kennedy D.N. (2023). A proposed human structural brain connectivity matrix in the center for morphometric analysis harvard-oxford atlas framework: a historical perspective and future direction for enhancing the precision of human structural connectivity with a novel neuroanatomical typology. Dev. Neurosci..

[68] Desikan R.S., Ségonne F., Fischl B., Quinn B.T., Dickerson B.C., Blacker D., Buckner R.L., Dale A.M., Maguire R.P., Hyman B.T. (2006). An automated labeling system for subdividing the human cerebral cortex on mri scans into gyral based regions of interest. Neuroimage.

[69] Wu Y., Hong Y., Feng Y., Shen D., Yap P.T. (2020). Mitigating gyral bias in cortical tractography via asymmetric fiber orientation distributions. Med. Image Anal..

[70] Johnson S.C. (1967). Hierarchical clustering schemes. Psychometrika.

[71] Davies D.L., Bouldin D.W. (1979). A cluster separation measure. IEEE Trans. Pattern Anal. Mach. Intell..

[72] Shi C., Wei B., Wei S., Wang W., Liu H., Liu J. (2021). A quantitative discriminant method of elbow point for the optimal number of clusters in clustering algorithm. EURASIP J. Wirel. Commun. Netw..

[73] Vázquez A., López-López N., Sánchez A., Houenou J., Poupon C., Mangin J.F., Hernández C., Guevara P. (2020). Ffclust: Fast fiber clustering for large tractography datasets for a detailed study of brain connectivity. Neuroimage.

[74] Hyvärinen A., Oja E. (2000). Independent component analysis: algorithms and applications. Neural Netw..

[75] Calhoun V.D., Liu J., Adalı T. (2009). A review of group ica for fmri data and ica for joint inference of imaging, genetic, and erp data. Neuroimage.

[76] Varoquaux G., Sadaghiani S., Pinel P., Kleinschmidt A., Poline J.B., Thirion B. (2010). A group model for stable multi-subject ica on fmri datasets. Neuroimage.

[77] Reeves W.D., Ahmed I., Jackson B.S., Sun W., Williams C.F., Davis C.L., Mcdowell J.E., Yanasak N.E., Su S., Zhao Q. (2025). fmri-based data-driven brain parcellation using independent component analysis. J. Neurosci. Methods.

[78] Basile G.A., Bertino S., Nozais V., Bramanti A., Ciurleo R., Anastasi G.P., Milardi D., Cacciola A.40 (2022). White matter substrates of functional connectivity dynamics in the human brain. Neuroimage.

[79] Basile G.A., Nozais V., Quartarone A., Giustiniani A., Ielo A., Cerasa A., Milardi D., Abdallah M., Thiebaut de Schotten M., Forkel S.J., Cacciola A. (2024). Functional anatomy and topographical organization of the frontotemporal arcuate fasciculus. Commun. Biol..

[80] Du Y., Fan Y. (2013). Group information guided ica for fmri data analysis. Neuroimage.

