# Supplementary material for: A high-resolution functional network-organized atlas of human superficial white matter from ultra-high-field diffusion MRI
Source: iScience. 2026 Jul 6;29(7):116671. doi: 10.1016/j.isci.2026.116671 (PMC13355829; doi:10.1016/j.isci.2026.116671)
Supplement: Document S1. Figures S1–S6 and Table S1 [file mmc1.pdf]

iScience, Volume *29*

Supplemental information

A high-resolution functional network-organized atlas of human superficial white matter from ultra-high-field diffusion MRI

Yifei He, Yu Xie, Hiuying Yip, Yoonmi Hong, and Ye Wu

Figure S1. Pairwise Dice overlap of thresholdQed TDI masks, related to STAR Methods.

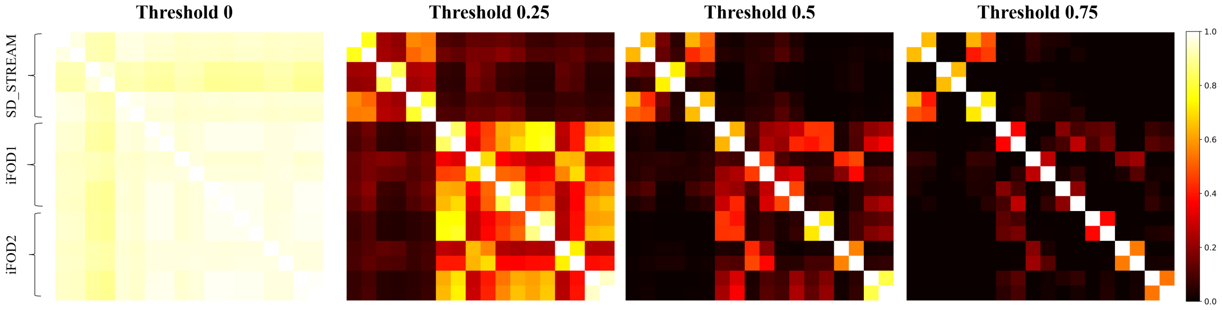

Figure S1. Pairwise overlap was quantified using the Dice coefficient between thresholded masks derived from whole-brain TDI maps generated by different tractography methods and parameter settings. Normalized TDIs were converted into binary masks using thresholds of 0, 0.25, 0.5, and 0.75. A Dice value closer to 1 indicates greater agreement between the corresponding masks. In each heatmap, rows and columns follow the same ordering from top to bottom and from left to right; each entry corresponds to a combination of a tractography algorithm and a parameter setting, arranged in the order SD_STREAM, iFOD1, and iFOD2. The masks derived at threshold 0 were nearly identical across methods, indicating broadly similar spatial coverage of the tractograms, whereas differences became more pronounced at higher thresholds, suggesting greater method-dependent variability in regions with relatively high track density.

Figure S2. Mean TDI maps across tractography methods, related to STAR Methods.

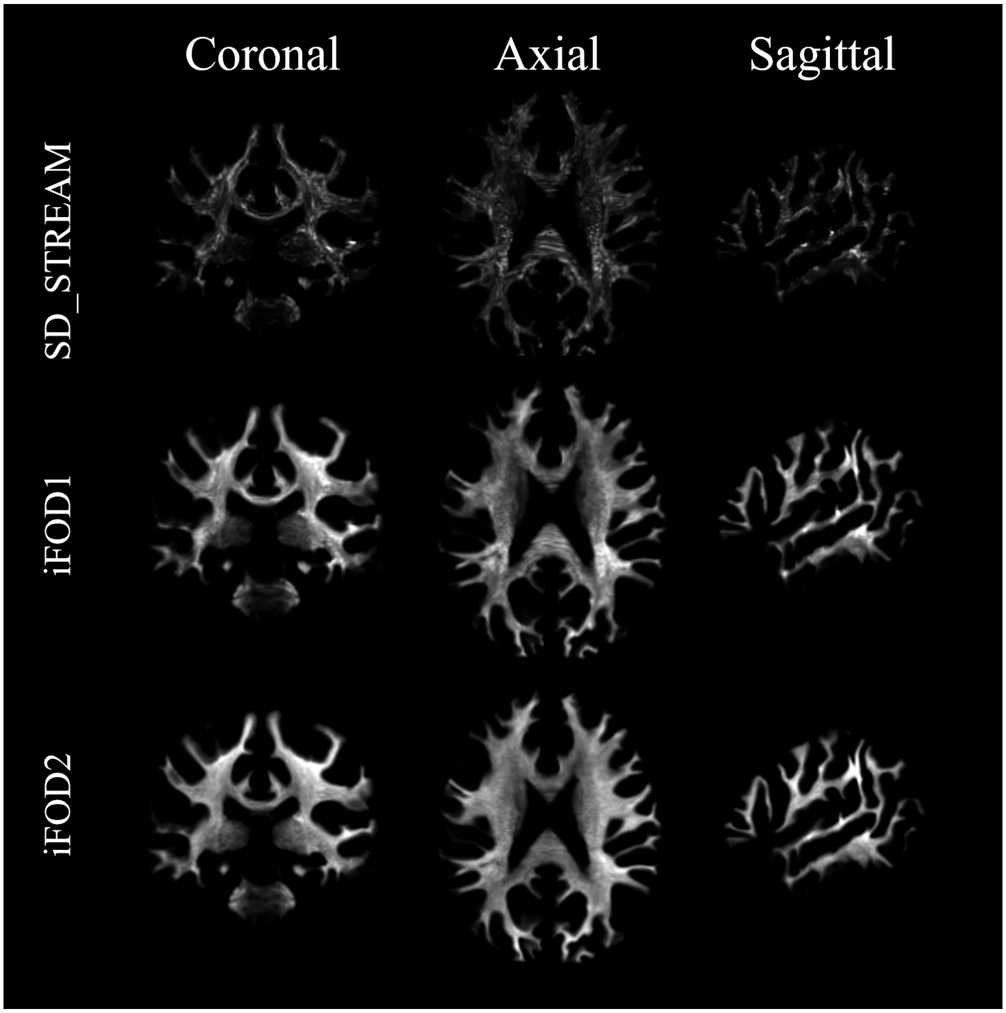

Figure S2. Mean normalized TDI maps are shown for the three tractography algorithms, including SD_STREAM, iFOD1, and iFOD2. For each algorithm, normalized TDI maps were averaged across all parameter settings to summarize the spatial distribution of reconstructed streamlines. Although the overall spatial distributions were broadly consistent across methods, local TDI intensities differed across algorithms, reflecting tractography-dependent variation in reconstructed fiber density. SD_STREAM showed more pronounced differences compared with the two probabilistic methods, iFOD1 and iFOD2. The displayed intensity range is 0 to 0.5.

Table S1. Variance explained in TW-dFC-derived uncertainty metrics by structural properties, related to STAR Methods.

Table S1: Summary of univariable and multivariable regression results for the strict and relaxed uncertainty metrics, showing the proportion of variance explained by fiber count, spatial span, and mean streamline length. Each variable was first tested separately in univariable models, and all three variables were then included jointly in a multivariable model. Values indicate the proportion of variance explained by each model, reported as R^2. All regression models were statistically significant $(p < 0.001)$.

Outcome	Fiber count R^2	Span R^2	Mean length R^2	Multivariable R^2
Strict uncertainty	0.033	0.100	0.144	0.183
Relaxed uncertainty	0.026	0.146	0.185	0.229

Figure S3. Regression trends of TW-dFC-derived uncertainty metrics, related to STAR Methods.

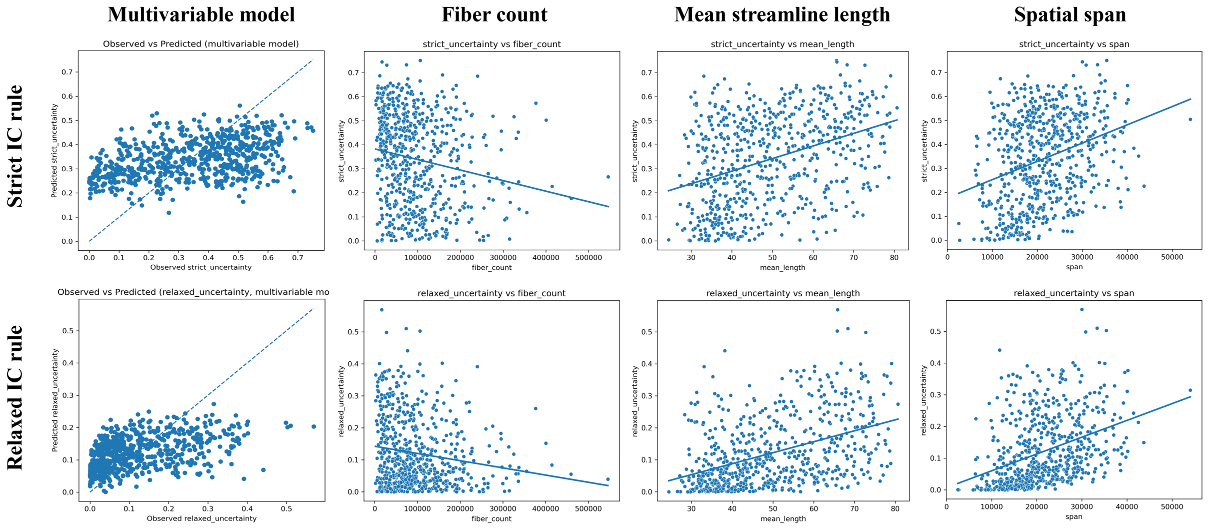

Figure S3. Regression analyses of the strict and relaxed uncertainty metrics against basic structural properties of SWM clusters. The top and bottom rows correspond to the strict and relaxed uncertainty measures, respectively. The first column shows the observed versus predicted values from the multivariable linear regression model including all three factors, and the other three columns show the univariable relationships with fiber count, mean streamline length, and spatial span.

Figure S4. Neurosynth decoding of DMN self-connection clusters, related to Figure 4.

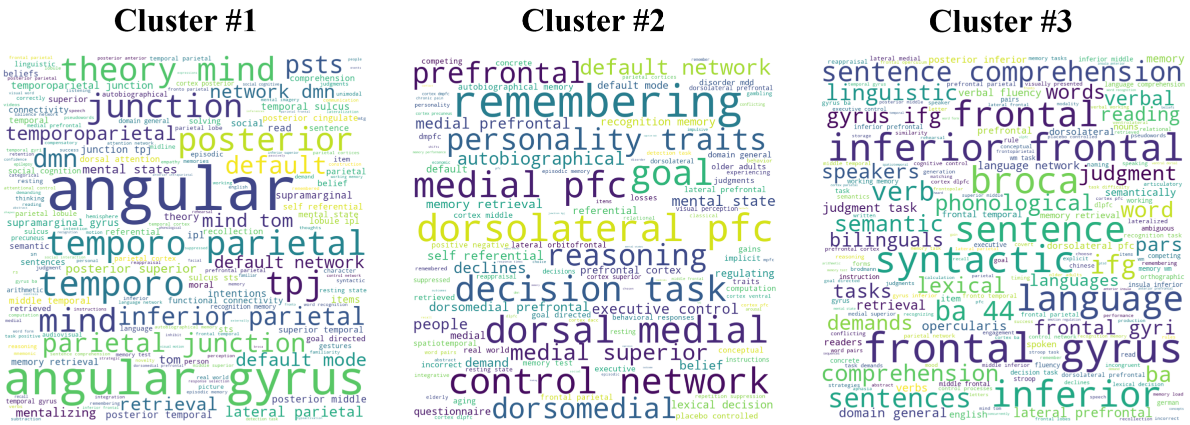

Figure S4. Word clouds of the Neurosynth decoding results for three fiber clusters corresponding to self-connections within the default mode network.

Figure S5. Yeo and DKT atlas adjacency matrices, related to STAR Methods.

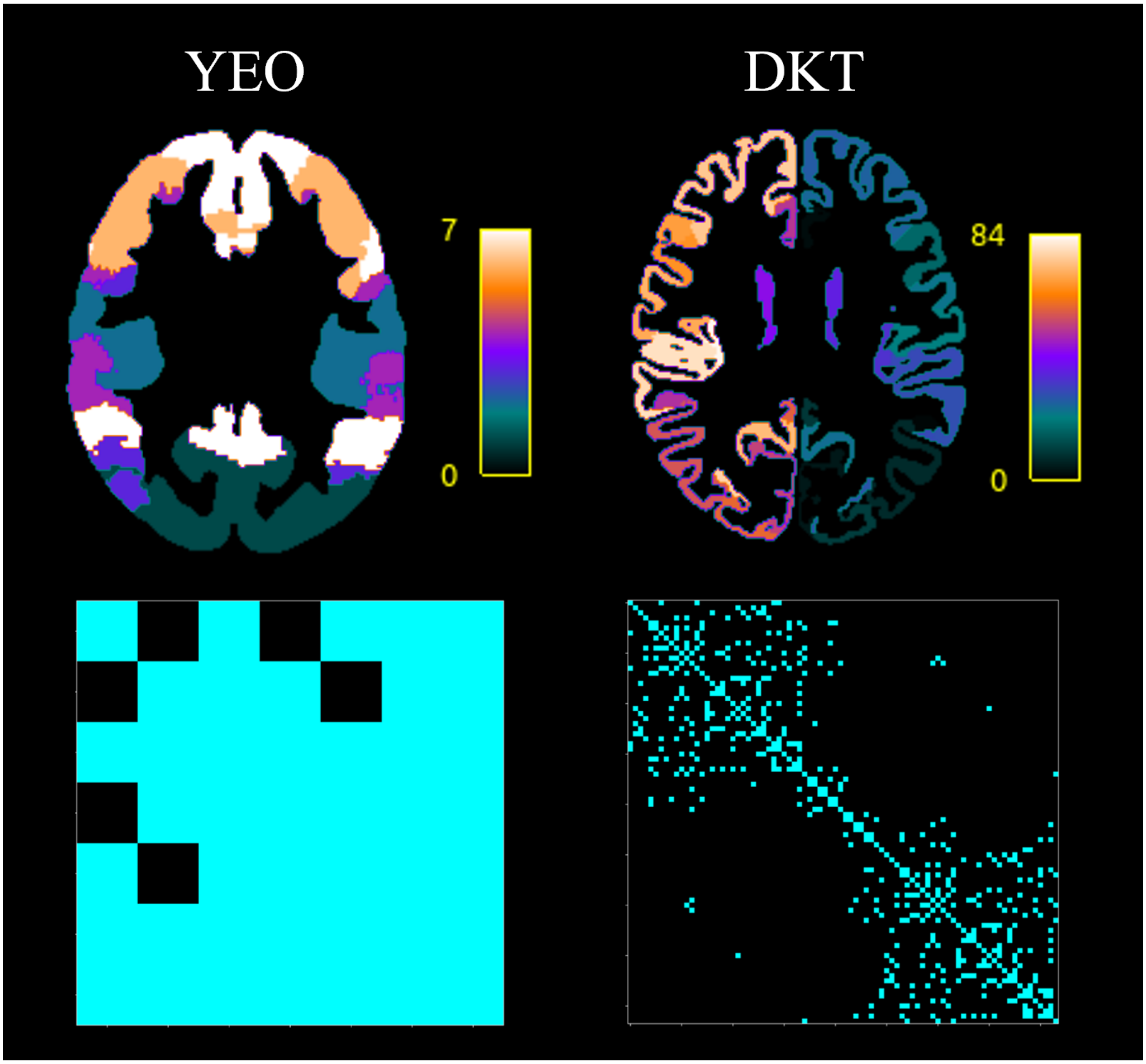

Figure S5. Comparison of the YEO and DKT atlases. Top: cortical parcellations. Bottom: corresponding adjacency matrices of cortical regions. Blue dots denote adjacent region pairs.

Figure S6. Representative SWM candidates excluded by the network classifier, related to STAR Methods.

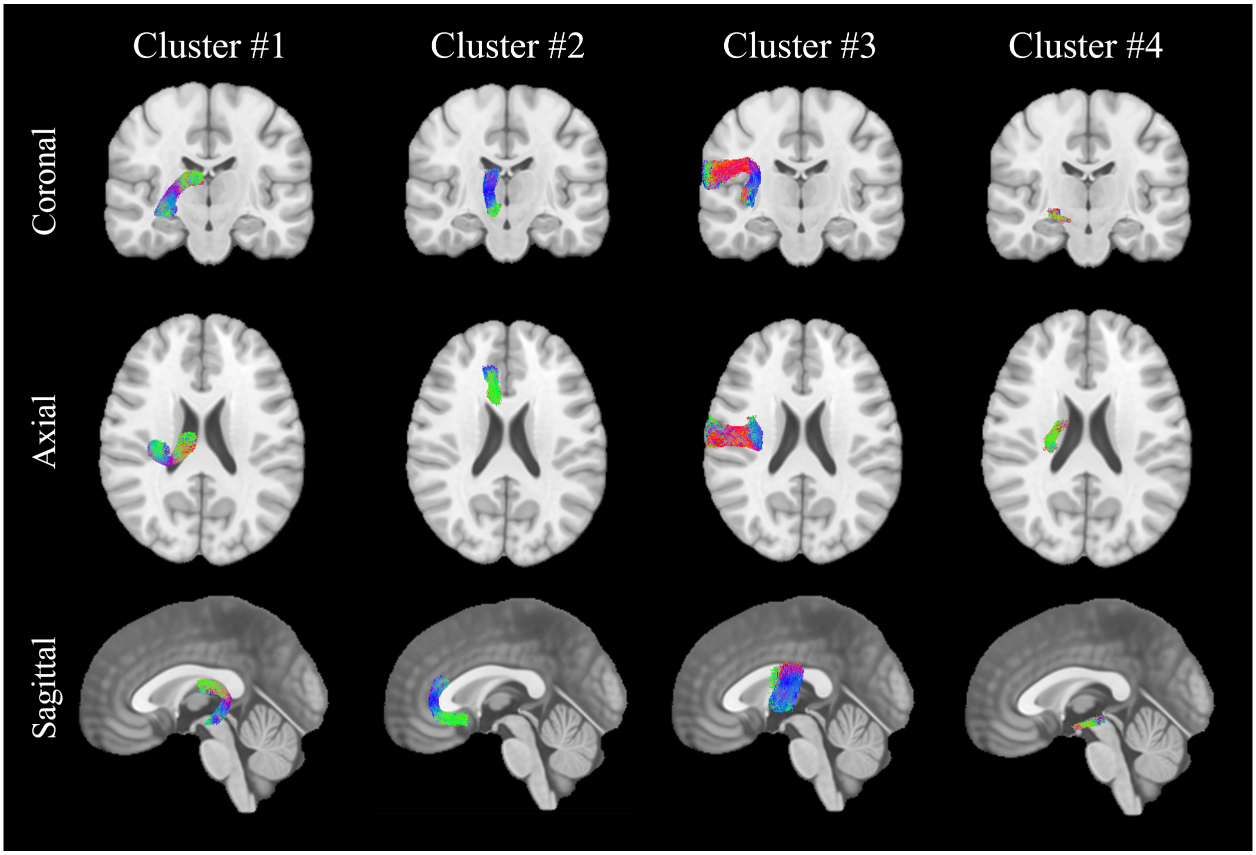

Figure S6. Representative fiber clusters that were identified as SWM by the shape- and position-based filters but were excluded by the network classifier.
